# Using matrix assisted laser desorption ionisation mass spectrometry combined with machine learning for vaccine authenticity screening

**DOI:** 10.1038/s41541-024-00946-5

**Published:** 2024-08-28

**Authors:** Rebecca Clarke, Tehmina Bharucha, Benediktus Yohan Arman, Bevin Gangadharan, Laura Gomez Fernandez, Sara Mosca, Qianqi Lin, Kerlijn Van Assche, Robert Stokes, Susanna Dunachie, Michael Deats, Hamid A. Merchant, Céline Caillet, John Walsby-Tickle, Fay Probert, Pavel Matousek, Paul N. Newton, Nicole Zitzmann, James S. O. McCullagh

**Affiliations:** 1 Department of Chemistry, University of Oxford, Oxford, OX1 3TA UK; 2 Department of Biochemistry, University of Oxford, Oxford, OX1 3QU UK; 3 Kavli Institute for Nanoscience Discovery, University of Oxford, Oxford, OX1 3QU UK; 4 Central Laser Facility, Research Complex at Harwell, STFC Rutherford Appleton Laboratory, UK Research and Innovation (UKRI), Harwell Campus, Didcot, OX11 0QX UK; 5 Medicine Quality Research Group, NDM Centre for Global Health Research, Nuffield Department of Medicine, University of Oxford, Oxford, OX3 7LG UK; 6 Mahidol-Oxford Tropical Medicine Research Unit, Faculty of Tropical Medicine, Mahidol University, Bangkok, 10400 Thailand; 7 Infectious Diseases Data Observatory, Nuffield Department of Medicine, University of Oxford, Oxford, OX3 7LG UK; 8 Agilent Technologies LDA UK, Didcot, OX11 0RA UK; 9 NDM Centre for Global Health Research, Nuffield Department of Medicine, University of Oxford, Oxford, OX3 7LG UK; 10 NIHR Oxford Biomedical Research Centre, Oxford University Hospitals NHS Foundation Trust, Oxford, OX3 9DU UK; 11 Department of Pharmacy, School of Applied Sciences, University of Huddersfield, Huddersfield, HD1 3DH UK; 12 Present Address: Hybrid Materials for Opto-Electronics Group, Department of Molecules and Materials, MESA+ Institute for Nanotechnology, Molecules Center and Center for Brain-Inspired Nano Systems, Faculty of Science and Technology, University of Twente, 7500AE Enschede, the Netherlands; 13 Present Address: Department of Bioscience, School of Health, Sport and Bioscience, University of East London, Water Lane, London, E15 4LZ UK

**Keywords:** Population screening, Adjuvants, Predictive markers, Drug regulation

## Abstract

The global population is increasingly reliant on vaccines to maintain population health with billions of doses used annually in immunisation programmes. Substandard and falsified vaccines are becoming more prevalent, caused by both the degradation of authentic vaccines but also deliberately falsified vaccine products. These threaten public health, and the increase in vaccine falsification is now a major concern. There is currently no coordinated global infrastructure or screening methods to monitor vaccine supply chains. In this study, we developed and validated a matrix-assisted laser desorption/ionisation-mass spectrometry (MALDI-MS) workflow that used open-source machine learning and statistical analysis to distinguish authentic and falsified vaccines. We validated the method on two different MALDI-MS instruments used worldwide for clinical applications. Our results show that multivariate data modelling and diagnostic mass spectra can be used to distinguish authentic and falsified vaccines providing proof-of-concept that MALDI-MS can be used as a screening tool to monitor vaccine supply chains.

## Introduction

Safe and effective medicines are crucial to people’s health worldwide but an increase in substandard and falsified pharmaceutical products threatens public health on a global scale. The World Health Organisation estimated that over 10% of pharmaceutical products in lower and middle-income countries were substandard or falsified (SF) in 2017 and has identified SF medicines as one of the urgent health challenges for the next decade^1,2^.

Reports of SF vaccine products have been increasing in recent years, including rabies, cholera, meningitis, yellow fever, hepatitis B and coronavirus disease 2019 (COVID-19). For example, in the first 15 months of the global COVID-19 vaccination programme, there were over 184 reports, across 48 countries, of diverted and SF COVID-19 vaccines, involving millions of doses^3^. A range of adulteration and falsification incidents have been identified, including replacement of vaccines with saline or other adjuvants such as sugar solutions and antibiotics, and errors in manufacture have led to substandard production^4–8^. Before the COVID-19 pandemic, there were multiple examples, including low potency rabies vaccines for dogs in China^9^, contaminated Salk polio vaccine in the USA^10^, falsified rabies vaccines in the Philippines^11^, falsified yellow fever vaccine in Bangladesh^12^ and mass administration by health workers of falsified routine childhood vaccines in Indonesia^13^.

Substandard vaccines arise from inadvertent errors in manufacturing and/or degradation in supply chains (e.g. inappropriate cold chain management), and falsified (aka counterfeited) vaccines arise from criminal, fraudulent activities^14^. It is important to distinguish these as the origins and solutions are different, but both are a major health risk for recipients with the potential to lead to increased morbidity and mortality and undermine the reputation of vaccines as safe medical products that play a vital role in maintaining the health of communities worldwide^15,16^. With a rise in vaccine use globally, it is becoming increasingly clear that a lack of risk analysis, monitoring and intervention within supply chains is allowing the problem of vaccine falsification, in particular, to develop^17,18^. The current lack of testing and monitoring represents a significant vulnerability, and new methods are required to enable risk-based post-market surveillance^2^. Vaccine supply chains are complex and rigorous testing at the proximal end of the supply chain, for example, will not mitigate against incidents downstream of this. Screening at the distal end of the supply chain may necessitate a larger, more differentiated testing network, spanning multiple locations and requiring rapid results. A range of techniques, devices and methods are therefore likely to be needed to effectively monitor supply chains for SF products and differentiate these from authentic vaccines^19^. Many, if not most, countries do not have laboratories able to check the quality of a diverse range of vaccines. Hence, testing methods are needed in central facilities that can rapidly give detailed information to facilitate decisions, ensuring that appropriate samples are sent to reference laboratories. Given the growing need for vaccine authenticity testing and the current lack of suitable methods, we explored matrix-assisted laser desorption/ionisation mass spectrometry (MALDI-MS) as an approach for detecting vaccine falsification.

Mass spectrometry (MS) has emerged as an important platform for molecular-level profiling, providing high sensitivity and high selectivity for the analysis of molecular composition in complex samples^20^. Machine learning and additional statistical approaches are also used to classify samples and identify biomarkers^21–24^. For example, metabolite profiles are used to differentiate healthy and disease states in biological extracts and blood products, such as serum and plasma, where machine learning is used to explore the large amounts of chemical information inherent in such datasets and implement *'*untargeted*'* hypothesis-generating approaches to data analysis^25–27^. Liquid chromatography–mass spectrometry (LC-MS) and gas chromatography–mass spectrometry (GC-MS) are commonly used for molecular characterisation but these research-grade instruments are expensive, require high levels of expertise to operate and are not evenly distributed worldwide, and therefore less favourable for screening at a global scale.

MALDI-MS is used in proteomics and, more recently, mass spectrometry imaging and molecular profiling applications such as metabolomics and small molecule pharmaceutical analysis^28–34^. Low sample volume requirements and the high-throughput nature of the analysis, provide significant benefits^35–39^. Recent developments in MALDI-MS applications for routine clinical testing are of specific interest; for example, used in high-throughput microorganism identification where pathogenic bacteria can be rapidly identified at low cost. The speed and effectiveness of this approach has led to worldwide deployment of MALDI-MS instruments; mainly Bruker MALDI Biotyper Sirius and bioMérieux VITEK MS systems in clinical laboratories for routine medical testing^40^. This provides an attractive, low-cost mass spectrometry platform with a global infrastructure that could be used for coordinated vaccine authenticity testing.

**Fig. 1:**
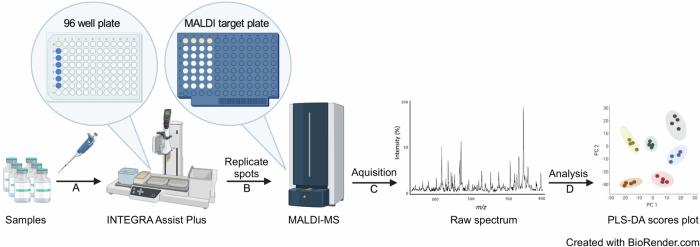
Matrix-assisted laser desorption/ionisation-mass spectrometry (MALDI-MS) sample preparation and analysis workflow. Step A: vaccine samples to be analysed are pipetted into a 96-well plate positioned in the INTEGRA Assist Plus. Step B: replicate spots of 1:1 (*V*/*V*) premixed sample and α-cyano-4-hydroxycinnamic acid (CHCA/HCCA) matrix are pipetted onto the target plates using the Assist Plus robot. Step C: raw spectra are acquired using the MALDI-MS instruments. Step D: data processing of the raw spectra and statistical analysis are performed. MALDI: matrix-assisted laser desorption/ionisation; PLS-DA partial least squares-discriminant analysis. This figure was created using BioRender.com.

Vaccines, depending on their type, can contain a wide range of antigens (as active ingredients), such as messenger RNAs (mRNAs), oligomers, viral vectors, live attenuated or killed organisms, lipids, polymers, proteins and a range of small molecule adjuvants which can include sugars and other biomolecules^41^. The heterogeneity of different vaccines, both in terms of diversity in active constituents, physiochemical properties and concentrations, makes samples challenging to characterise from an analytical perspective. To date, we are not aware of any applications using MALDI-MS for vaccine characterisation and authentication studies but the inherent sensitivity and molecular selectivity of MALDI-MS, and the existing worldwide availability of instrumentation in clinical microbiology laboratories, provides a compelling case to explore its potential as a device for vaccine authentication. The focus of this study was to explore the capabilities of MALDI-MS *“*biotyping*”* systems for vaccine analysis by developing a method and validating it for the analysis of authentic vaccine samples, falsified vaccines and their categorisation using machine learning approaches. For the data analysis, we explored several data processing software approaches, including SpectralWorks AnalyzerPro XD software which was then successfully used for processing and statistical analysis of the MALDI data. However, we found the open-source packages MALDIquant^42^ and MetaboAnalyst 5.0 webtool^43^ highly effective in combination and used these for the data analysis reported in this study. We tested the workflow using four different commercially available vaccines and a range of known-falsified vaccine compositions. We used machine learning and additional statistical analysis to model the data and predict *m/z* features from the experimental data that had the potential to be used in an online database approach for vaccine authenticity screening. Figure 1 provides a conceptual overview of the workflow developed in this study.

## Results

### Analysis of vaccines and falsified constituents by MALDI-MS

Four different authentic, commercially available, vaccines and eight falsified surrogates previously reported in falsified vaccine products^3^, were used in this study. The authentic vaccines were Nimenrix (Pfizer Ltd, Sandwich, UK), a conjugate vaccine that protects against *Neisseria meningitidis* groups A, C, W-135 and Y; Engerix B (GlaxoSmithKline, Brentford, UK), which protects against hepatitis B virus infection (HBV); Flucelvax Tetra (Seqirus Ltd., Maidenhead, UK) which protects against influenza (Sept/Oct 2021 to early 2022 season) and Ixiaro (Valneva Ltd., Fleet, UK), for immunisation against Japanese encephalitis virus infection. Information about genuine vaccines and falsified vaccine surrogates is provided in Table 1^3,8,44–49^.

**Table 1:** Samples used for analysis

Vaccine/falsified surrogate	Target pathogen/disease	Refs.
Engerix B	Hepatitis B infection	^44^
Flucelvax Tetra	Influenza virus strains	^45^
Ixiaro	Japanese encephalitis virus	^46^
Nimenrix	Meningococcal disease	^47^
Amikacin 250 mg/mL	−	^3^
Milli-Q water	−	^3^
Tap water	−	^3^
Water for injection	−	^3^
0.9% *m/V* sodium chloride	−	^3,48^
5% *m/V* glucose solution	−	^8^
Gentamicin 40 mg/mL	−	^48^
Hyaluronic acid	−	^49^

Genuine vaccines (including target pathogen/disease) and falsified vaccine constituents (including refs.) either previously reported to have been used or could be used as falsified vaccines.

**Fig. 2:**
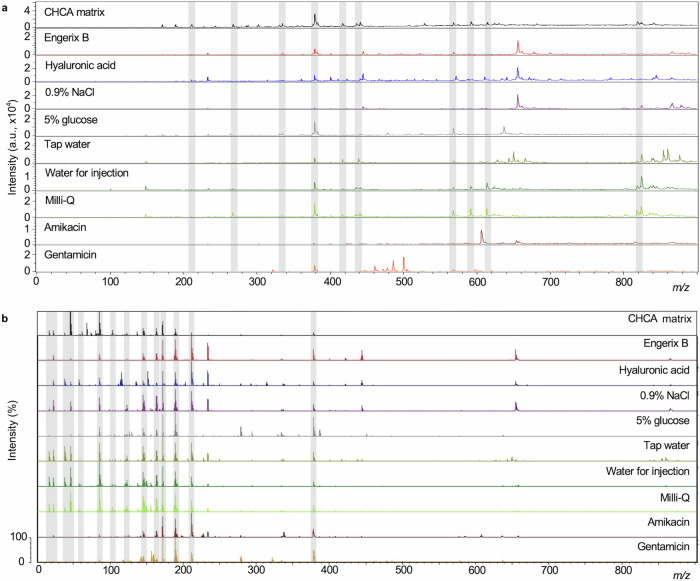
Representative mass spectra (*m/z* 0–900) for α-cyano-4-hydroxycinnamic acid (CHCA) matrix, Engerix B vaccine and eight samples of other compounds and mixtures previously reported as being constituents of falsified vaccines. **a** Biotyper Sirius mass spectra. **b** VITEK mass spectrometry (MS) spectra. Through the presence, absence and relative intensity ratios of peaks in the spectra, the genuine vaccine can be distinguished from the falsified constituents by manual inspection of spectra. Common matrix peaks are indicated by shaded bars.

**Fig. 3:**
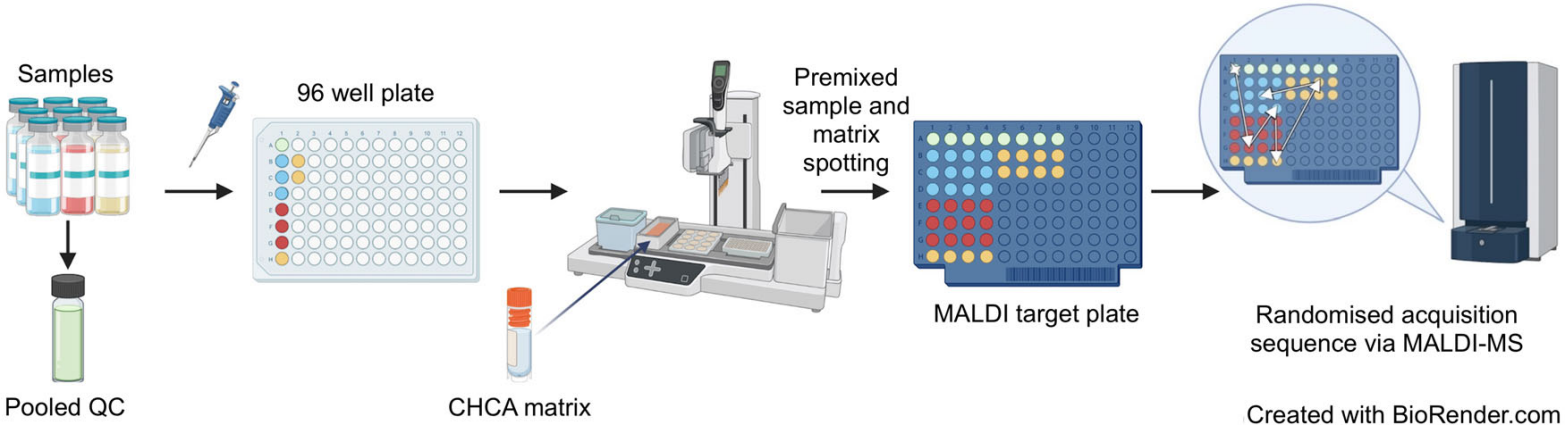
Schema showing samples and a pooled quality control (QC) sample being spotted onto a matrix-assisted laser desorption/ionisation (MALDI) target. A pooled QC sample was prepared from the vaccines and falsified samples. An Assist Plus robot was used to combine the matrix with each sample in a 1:1 (V/V) ratio and then spot onto the MALDI plate. Only the QC and first three samples are illustrated, but all four authentic vaccines and eight falsified constituent samples were prepared in the same way across multiple MALDI plates which were analysed in a random sequence within the MALDI instruments. CHCA: α-cyano-4-hydroxycinnamic acid; MALDI-MS: matrix-assisted laser desorption/ionisation-mass spectrometry. This figure was created using BioRender.com.

We performed sample analysis in parallel on two separate MALDI-MS systems, both routinely used for microorganism clinical testing with worldwide deployment. A MALDI Biotyper Sirius (Bruker Daltonics) and a VITEK MS (bioMérieux, Craponne, France). The two instruments provided very similar performance when combined with data modelling but interestingly provided slightly different mass spectral profiles when visually compared. First, we acquired mass spectra using methods adapted from the standard in vitro diagnostic (IVD) parameters provided on both instruments. We made slight adjustments to the laser raster pattern and percentage energy range to accommodate a broader range of sample types. Spectra were acquired over three different overlapping *m/z* ranges: 0–900; 700–2500 and 2000–20,000. Representative spectra for Engerix B and the eight falsified constituent samples at *m/z* 700–2500 and *m/z* 2000–20,000 mass ranges are shown in Supplementary Figs. 1, 2 for the Biotyper Sirius and VITEK MS instruments, respectively. Visible peaks in the low-mass range included matrix peaks that were common to all samples and could be identified from matrix blanks, as well as analyte peaks related to the individual samples. Given the rich spectral data obtained in the *m/z* 0–900 range, where vaccine-specific excipients were found, we decided to focus on this *m/z* range in further analyses. Figure 2 shows representative mass spectra for the Engerix B vaccine and each of the surrogate falsified samples as well as blank CHCA matrix at the *m/z* 0–900 range (similar comparisons for the other vaccines are provided in Supplementary Figures 3 & 4). Non-matrix peaks, that were unique to either individual vaccines or falsified constituents, were identified by manual inspection of the spectra. The spectral peaks in Fig. 3a, b provide an illustration of the presence and absence of mass spectral peaks which were observed for Engerix B and the falsified vaccine constituents. These analyses established the proof-of-principle that the MALDI-MS systems were capable of measuring mass spectral peaks that can distinguish genuine comparator vaccines from falsified vaccine surrogates.

### MALDI method development and validation

Having established the feasibility of distinguishing vaccines and falsified constituents by manual inspection, we next developed and validated a method and workflow for data processing and analysis. The reproducibility of MALDI-MS mass spectra is known to be largely affected by matrix type, sample composition and matrix-sample crystallisation conditions, as well as the specific laser ablation parameters^50–52^. We, therefore, investigated analytical reproducibility on both platforms.

In order to determine analytical *“*spot-to-spot*”* reproducibility and intra-batch (vaccine vial-to-vial) reproducibility, we analysed replicates of the four authentic vaccine samples and eight falsified surrogates. For each sample vial, we created four replicate spots on the MALDI target plate and replicated this three times using three separate vials (same manufacturer batch number/part number), so there were 12 MALDI sample spots for each vaccine and falsified constituent on a MALDI plate. All samples were distributed across three Bruker MALDI plates and six bioMérieux MALDI slides, respectively (due to the different dimensions of the plates for both systems). We also created a pooled quality control sample which comprised an equal volume mixture of each of the four authentic vaccines and eight falsified vaccine samples. The experiment was designed to investigate analytical reproducibility, spot-to-spot variability and vial-to-vial reproducibility. A schematic illustrating how the MALDI plate samples were spotted, and the plates configured is shown in Fig. 3.

**Fig. 4:**
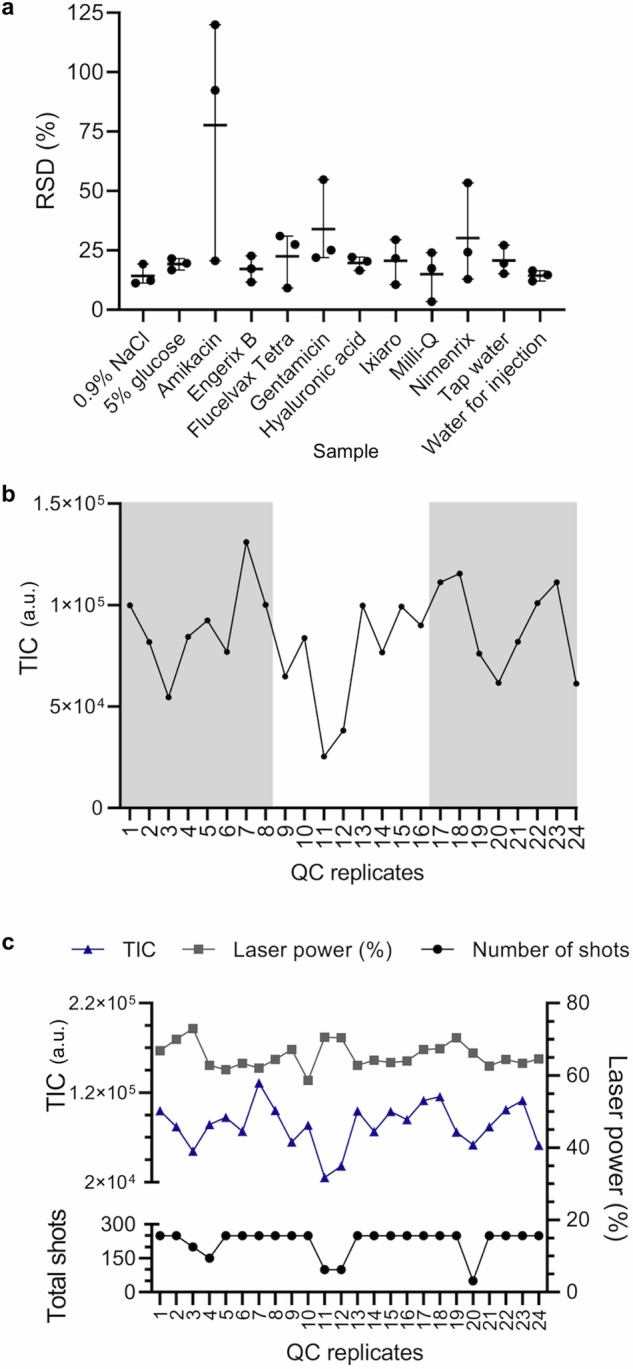
Method validation using mass spectrometry data. **a** The percentage relative standard deviation (RSD) values for each vial per sample are plotted showing the range and mean. **b** The total ion count (TIC) for each quality control (QC) sample replicate plotted in consecutive run order shows no particular bias (replicates spotted on different target plates are alternately shaded/white). **c** TIC, laser power, and number of shots of the laser for replicates plotted consecutively for each QC sample.

Each MALDI spot was analysed under the same settings for each instrument. A randomised acquisition sequence was used to control for any bias in sample preparation or run order. Table 2 provides the percentage RSD for the total ion intensity for all 12 replicates of each sample and 24 QC replicates prior to intensity calibration from analysis on the Sirius MALDI platform (equivalent data for the VITEK is given in Supplementary Table 1). These results show the total variation of the vaccine or falsified constituent samples. The range in RSD values for all samples except Amikacin was from 18 to 44% over all sample replicates for each group. This reproducibility in signal intensity was similar to the RSDs reported in other MALDI-based profiling studies using other sample types^53^. Figure 4a shows the vial-to-vial reproducibility specifically (e.g., inter-vial variability) for each genuine vaccine and falsified constituent, comprising individual percentage RSD calculations for the four sample preparation replicates of each vial. Equivalent data for the VITEK is shown in Supplementary Fig. 5a.

**Table 2:** Evaluated reproducibility of the raw data from the Biotyper Sirius (0–900 *m/z*)

Sample	RSD (%)
0.9% *m/V* sodium chloride	17.95
5% *m/V* glucose	26.17
Amikacin 250 mg/mL	122.51
Gentamicin 40 mg/mL	41.83
Hyaluronic acid	22.18
Tap water	23.59
Milli-Q water	19.94
Water for injection	18.01
Engerix B	19.58
Flucelvax Tetra	26.90
Ixiaro	22.75
Nimenrix	43.65
Quality control	28.75

The percentage relative standard deviation (RSD) values calculated from the total ion intensities of all 12 sample replicates and 24 quality control replicates are given.

Analysis of Amikacin, Gentamicin, and Nimenrix gave some of the highest RSD values and the total RSD for all 12 replicates of Amikacin was anomalously high at 122% in the Sirius data (see Table 2). These higher percentage RSD values correlated with poorer co-crystallisation of the sample with the CHCA matrix on the MALDI plate prior to analysis. For these three samples, all 12 replicates exhibited a shiny appearance on the spot surface as opposed to appearing matte with visible matrix crystals observed for most other samples. For Amikacin, the dried spots maintained a droplet-like three-dimensional structure (unlike all other samples which dried flat) and may have resulted in poor sample ionisation and, subsequently, greater intensity variation reflected in the percentage RSD values. This demonstrates the importance of ensuring optimal sample-matrix crystallisation conditions.

To investigate whether there was any observable bias in the intensity measurements, we next plotted the relationship between run order and peak intensity across the QC samples. Figure 4b illustrates the result from the Sirius showing no observable bias (similar results were obtained from the VITEK shown in Supplementary Fig. 5b). This suggested the process of analysing the MALDI plate in the ion source does not lead to bias in intensity measurement over time. Finally, in order to establish whether the variability observed in replicates of intensity measurements (indicated by the RSD values) was influenced by the laser power or the number of times the laser was fired, we plotted the laser power of the last 50 shots acquired (in the analysis of each sample spot) against the corresponding TICs and the total number of accumulated shots for each replicate in run order for the QC samples for the Bruker Sirius analysis (Fig. 4c). No correlation was observed suggesting total signal intensity was not biased by any variation in the laser power or in the number of laser firings that may occur between the analysis of different spots.

### Developing a data processing and analysis workflow using MALDIquant

**Fig. 5:**
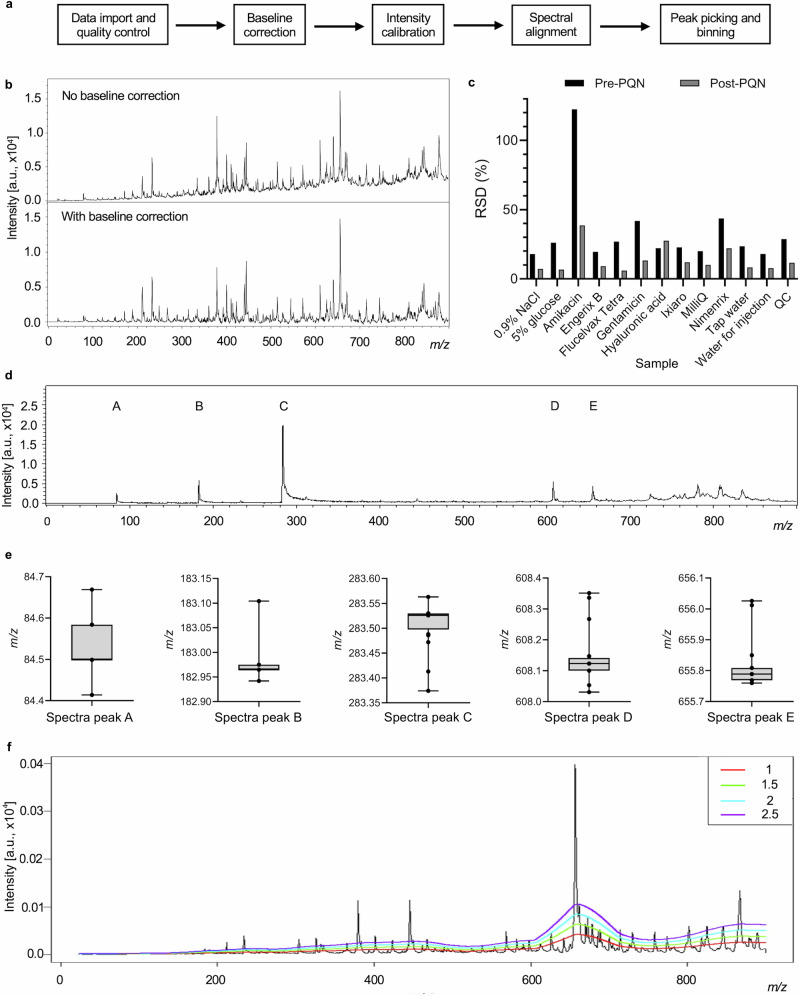
Data processing steps using MALDIquant (Bruker Biotyper Sirius data). **a** MALDIquant workflow. **b** Baseline drift correction using TopHat algorithm, spectra for hyaluronic acid. **c** Comparing the effect of pre and post probabilistic quotient normalisation (PQN) on the percentage relative standard deviation (RSD) for the vaccine, falsified constituent, and quality control (QC) sample replicates. **d** QC spectrum showing peaks labelled A–E used to illustrate *m/z* variation. **e** Box plots illustrating variation in *m/z* across 24 QC samples for peaks labelled A–E in part D. The line in the grey box indicates the median value, with the box limits showing the interquartile range. Whiskers extend to max and min values. **f** Comparing different signal-to-noise ratio (SNR) thresholds using an averaged mass spectrum incorporating authentic and falsified vaccines/constituents. Coloured coded numbering representing SNR thresholds.

After establishing that multiple authentic and falsified vaccine constituents could be reproducibly differentiated by the identification of unique mass spectral peaks, and having established reproducibility of peak intensities across replicate samples, we next developed a spectral data processing workflow using the MALDIquant R package. Figure 5a illustrates the main steps in the workflow developed. This includes combining the full spectrum data from all samples into a table for each replicate across all samples, baseline correction, peak intensity normalisation and peak identification. These steps were performed to reduce experimental and analytical variability in the dataset, and to align peaks and their intensities between samples. To do this, we evaluated each step using our vaccine and falsified vaccine sample dataset. The data processing was performed using data from both MALDI platforms. Spectra files were imported into R in mzXML format, with quality control by visual inspection.

Baseline drift across the mass range is a common feature of MALDI-mass spectra, and this can interfere with peak intensity comparisons between samples. For example, in Fig. 5b the upper spectrum without correction shows the baseline drifts with increasing *m/z*. MALDIquant provides either a statistics-sensitive non-linear iterative peak-clipping (SNIP) algorithm developed by Ryan et al.^54^, a TopHat approach derived from mathematical morphology^55^, ConvexHull or median algorithm to correct for this, based on user selection. We applied the TopHat baseline correction to each acquired spectrum which mimicked the default algorithm set in Bruker flexControl software. The lower mass spectrum in Fig. 5b shows the result of applying the baseline correction with the beneficial effect of lowering the baseline, especially towards the higher end of the mass range.

**Fig. 6:**
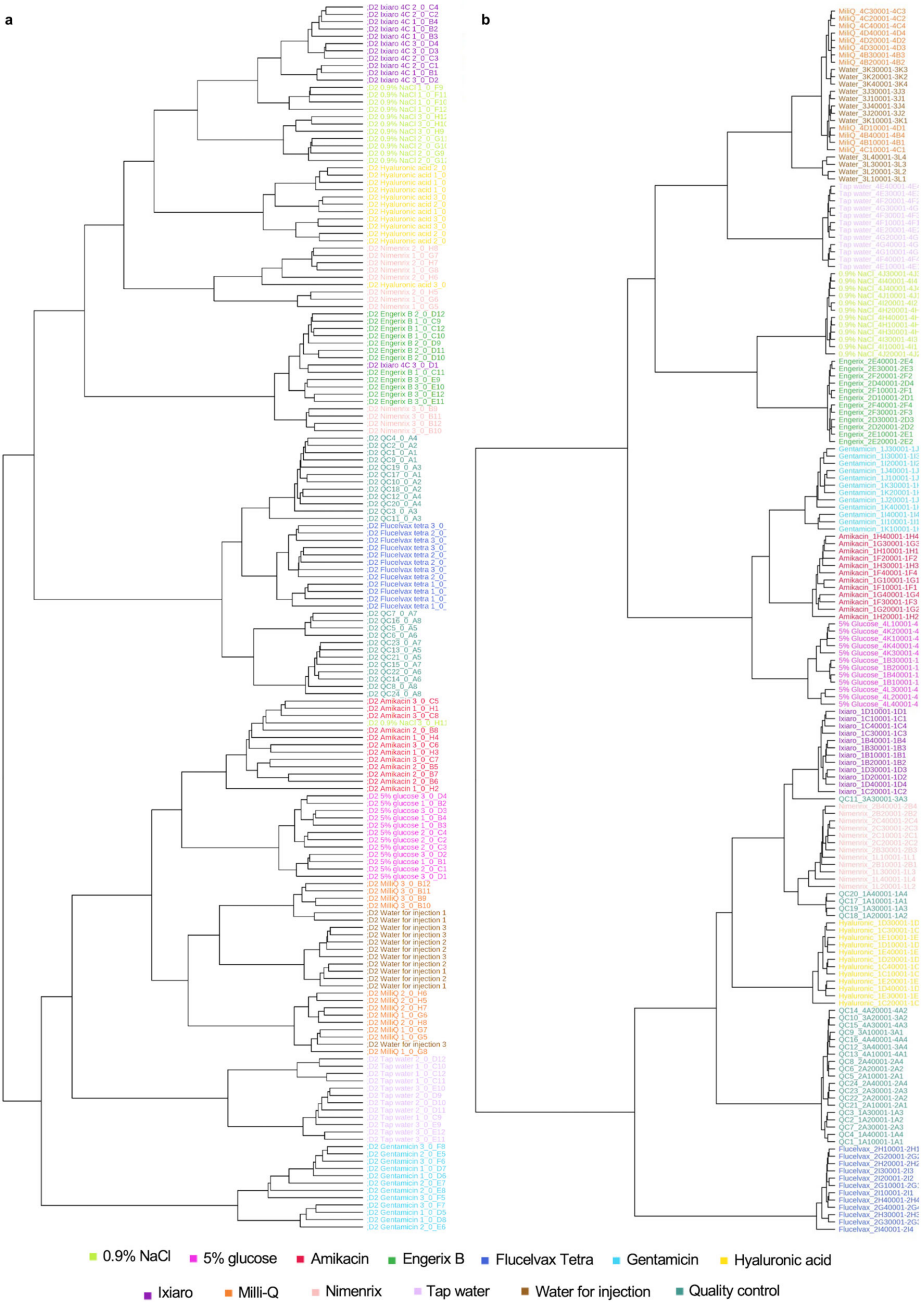
Multivariate statistical analysis discriminates the authentic vaccines Engerix B, Flucelvax Tetra, Ixiaro and Nimenrix from falsified vaccine constituents. **a** Biotyper Sirius dendrogram. **b** VITEK MS dendrogram. Hierarchical clustering dendrogram of all samples sorts almost all sample replicates (*n* = 12 for each sample type) into their respective groups.

Intensity shifts from one replicate spectrum to another were identified in the analysis of the vaccine and falsified constituent samples (see sample RSD variation in Fig. 6a, b and QC sample analysis in Fig. 5c). Post-acquisition data normalisation can be used to minimise these variations and reduce the influence of experimental or analytical variability. There are various statistical approaches (used extensively in metabolomics, for example) where large datasets are compared, and here a probabilistic quotient normalisation (PQN) was applied^56^. This was found to have a positive effect by lowering the RSD values in almost all cases (Fig. 5c).

After data normalisation, variations in *m/z* were evaluated and corrected to ensure effective comparisons could be made across multiple samples in the experiment. Figure 5d shows a representative mass spectrum of the QC sample with five peaks labelled (A − E). Peaks A to E in Fig. 5d show a variation in *m/z* across the 24 QC replicates which are illustrated by the box plots in Fig. 5e. The mean average range in *m/z* value per peak was 0.231 Da with a standard deviation of 0.06 Da. This variability is largely due to differences in peak shape where flat top peaks lead to fluctuation in the centroided *m/z* value (Exemplar peak shapes shown in Supplementary Fig. 6). Peaks were aligned to correct for this using non-linear warping of peaks with the locally weighted scatterplot smoothing (LOWESS) method^57,58^ with tolerance, SNR and half-window size parameters selected to optimise the spectral alignment of the dataset.

To evaluate how mass spectral peaks are *'*picked*'*, (e.g. automatically recognised as an individual mass spectral peak) and accurately assigned across samples, we tested various signal-to-noise ratio threshold settings. MALDIquant can identify local maxima and minima across the mass spectrum and then compare which peaks are above a set SNR threshold to identify the signal as a spectral peak for inclusion in the dataset. Figure 5f illustrates the effect of different signal-to-noise ratios using an averaged mass spectrum of all the genuine and falsified vaccine samples. Peak binning (with a user-defined threshold) was also used at this stage to ensure individual *m/z* features were correctly assigned across all the mass spectra. This increases mass spectral precision to ensure a more effective data comparison. The threshold for peak binning was chosen based on an evaluation of the spectral resolution across the dataset.

### Vaccine authentication using machine learning (ML)

**Fig. 7:**
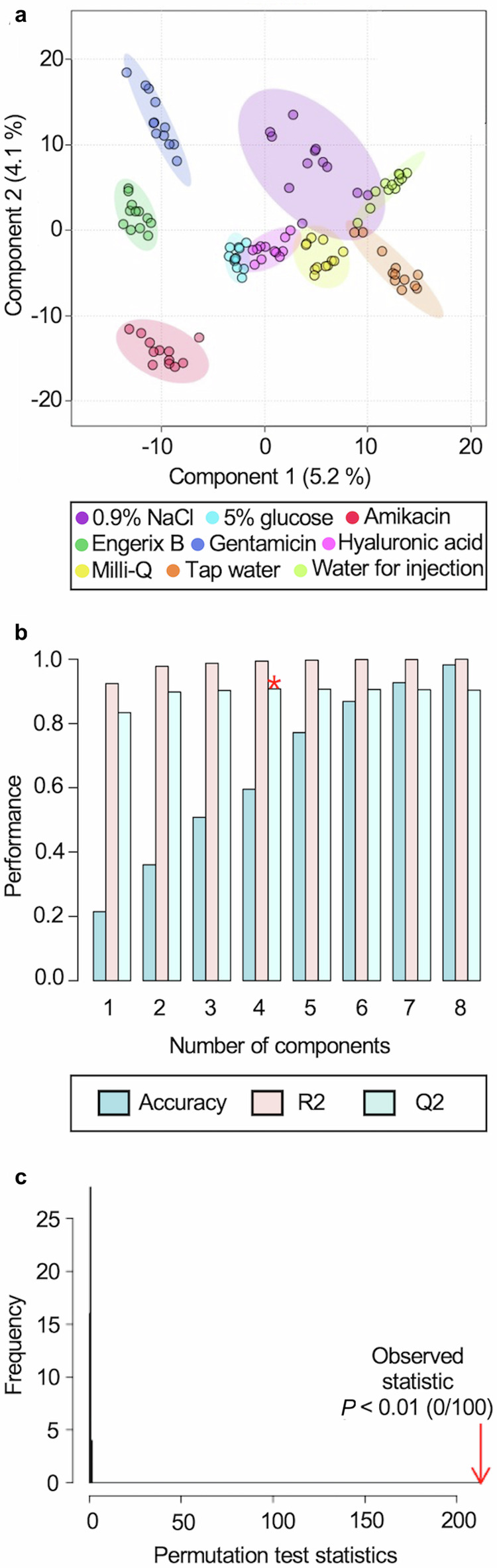
Biotyper Sirius partial least squares-discriminant analysis (PLS-DA) of authentic vaccine Engerix B and all falsified vaccine constituents, m/z 0–900. **a** PLS-DA two-dimensional scores plot shows sample group clustering. **b** Cross-validation shows a minimum of four components (mass spectral peaks) are required to differentiate the experimental groups for the best Q-squared (Q2) value (shown by *). Supplementary Table 3 gives the numerical values for the performance of accuracy, R-squared (R2) and Q2 in the cross-validation. The performance axis indicates the predictive ability of the model. **c** Permutation testing showed the model was significant with *P* < 0.01.

Having developed and validated a combined sample analysis and data processing workflow we applied this to analyse and compare authentic and falsified vaccine constituents using both MALDI platforms in parallel. We analysed samples from three replicate vials of each of the four authentic vaccines and eight falsified vaccine surrogates. Four analytical replicates were also analysed for each vial replicate to investigate analytical and vaccine vial-to-vial reproducibility. The samples were spotted and then analysed using the 0–900 *m/z* range. The resulting data were processed using the MALDIquant workflow developed, and a data table representing all the results was produced (example given in Supplementary Table 2). The heatmap in Supplementary Fig. 7 provides a visual overview of the dataset and was used to confirm that no individual or experimental class outliers were present (equivalent figure for the VITEK MS in Supplementary Fig. 8). To explore whether the vaccines and falsified constituents could be distinguished from each other using a multivariate statistical machine learning approach, we first performed hierarchical clustering (based on a Euclidean distance measure and a Ward clustering algorithm). We found that each of the samples replicates clustered together (Fig. 6) in almost all cases for the data collected on both MALDI platforms, which showed that both datasets contained *m/z* features that could differentiate authentic and falsified vaccines. To statistically model how well the data could distinguish the different sample groups, we compared each individual authentic vaccine with all the falsified vaccine samples using partial least squares-discriminant analysis (PLS-DA), commonly used in untargeted data modelling^59,60^. PLS-DA is a supervised dimensionality reduction method that builds models based on input variables and identifies which of these variables maximise separation between the groups. Validated models can be used to make future predictions on new data presented to the model. We first created a PLS-DA model using the Biotyper Sirius data for the authentic Engerix B vaccine with all the falsified vaccines. To illustrate the results, the PLS-DA scores plot (Fig. 7a) shows sample replicates cluster by sample type, and the model distinguished the authentic vaccine from the falsified vaccine constituents (and also the falsified constituents from each other) and was shown to create a strong model that was not overfitting the data (Fig. 7b, c). We subsequently created models for each authentic vaccine using both the Sirius and VITEK datasets. To demonstrate that the PLS-DA models were reliable and not overfitting the datasets, we performed cross-validation, permutation testing and a modified external validation for each model^61^. For the Engerix B Sirius data model R-squared (R2) and Q-squared (Q2) were between 0.8 and 1 and the permutation test statistic was *P* < 0.01 (Fig. 7b, c)^62^. Tabulated values for the PLS-DA cross-validation are displayed in Supplementary Table 3 (and the equivalent PLS-DA plots for the VITEK Engerix B data are shown in Supplementary Fig. 9). Similar results were obtained when comparing the other three genuine vaccines with all falsified vaccine surrogates across both MALDI platforms (Supplementary Figs. 10–15). We also performed an independent external validation where each dataset was randomly split into a training set (80% of the data) and an external test set (20% of the data). The models were created using the training set, and then the classifications were confirmed using the test set (which had not been seen by the model previously). Confusion matrices (see Supplementary Tables 4–27, with the genuine vaccine highlighted in yellow) were created for the external validation datasets, and in each case (for both Sirius and VITEK results), the authentic vaccines were predicted correctly^63^. In some cases, the different types of water and saline falsified constituents were not fully resolved, but this was not unexpected considering their compositional similarity and this did not compromise the identification of the authentic vaccines. In summary, our PLS-DA modelling demonstrated that the MALDI-MS data could be used to reliably predict each genuine vaccine from falsified constituents.

**Fig. 8:**
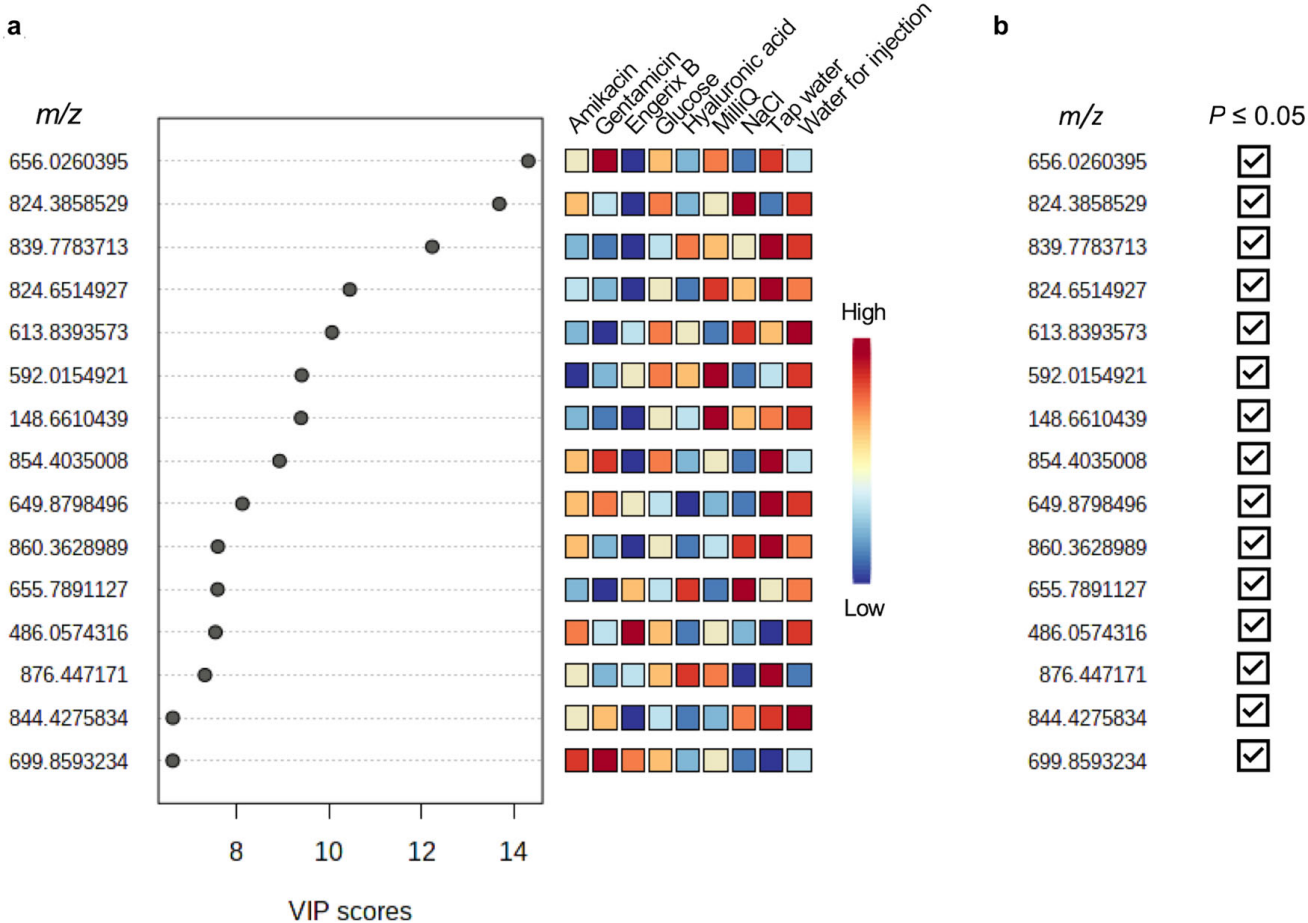
Biotyper Sirius analyses of compound feature significance. **a** Variable importance in the projection (VIP) of the peaks at *m/z* 0–900 for the Engerix B vaccine compared to the eight falsified constituents. The top 15 *m/z* values are plotted based on their VIP score. The heatmaps to the right of the plot represent the relative intensities of the *m/z* values for each sample group averaged over the group. **b** Two-way analysis of variance (ANOVA) with Dunnett multiple comparison test results for the top 15 *m/z* values from the VIP analysis. *m/z* values with at least one statistically significant comparison (*P* < 0.05) for a falsified constituent compared to Engerix B are marked with a check.

Next, we identified the most discriminatory mass spectral peaks in the models by examining the top 15 *m/z* features in the Variable Importance in the Projection (VIP) plot. Figure 8a shows the ranking of each of the top 15 *m/z* values from the Sirius data by way of example. The mass spectral abundance differences for the top 15 VIPs were statistically significant for at least one or more of the falsified constituents individually compared to Engerix B (two-way ANOVA with Dunnett multiple comparison test, Fig. 8b). Supplementary Figs. 16–22 further illustrate Sirius and VITEK MS VIP plots and ANOVA summaries for the falsified surrogates compared to the genuine vaccines. The PLS-DA results demonstrated that the MALDI data modelling, based on the full MALDI-mass spectrum, could be used to discriminate between authentic vaccines and falsified vaccine constituents in addition to the four genuine vaccines themselves (Supplementary Fig. 23).

One way to implement the MALDI-MS method as a tool for vaccine supply chain screening, would be to automate matching and scoring multiple spectral peaks identified in experimental samples with an online database containing multiple discriminatory *m/z* features previously collected and validated using samples of authentic vaccines. For example, a real-time score or percentage match for the mass spectral profile could be used to indicate the likelihood of vaccine authenticity. This approach is analogous to that currently used for bacterial strain identification by MALDI-MS in clinical laboratories worldwide. A complex profile of multiple *m/z* features would, therefore, be required to make a positive match with a falsified product and creating such a falsified product with the necessary specificity would likely be impractical and uneconomic.

**Fig. 9:**
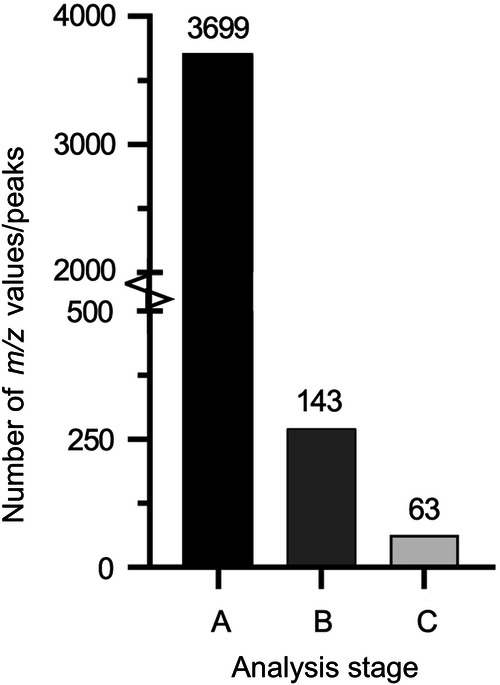
Bar graph of the numbers of *m/z* values and spectral peaks following two-way analysis of variance with Dunnett multiple comparison test between Engerix B and the falsified vaccine surrogates for Biotyper Sirius data. Bar A represents the 3699 total *m/z* values identified by MALDIquant peak detection and binning. B represents the 143 peaks in the raw spectra that yielded a statistically significant *P* value *(P* ≤ 0.05) for at least one falsified constituent compared to Engerix B. Bar C represents the 63 significant peaks in the raw spectra that have a clear presence in Engerix B and absence in at least one falsified constituent (or vice versa).

**Fig. 10:**
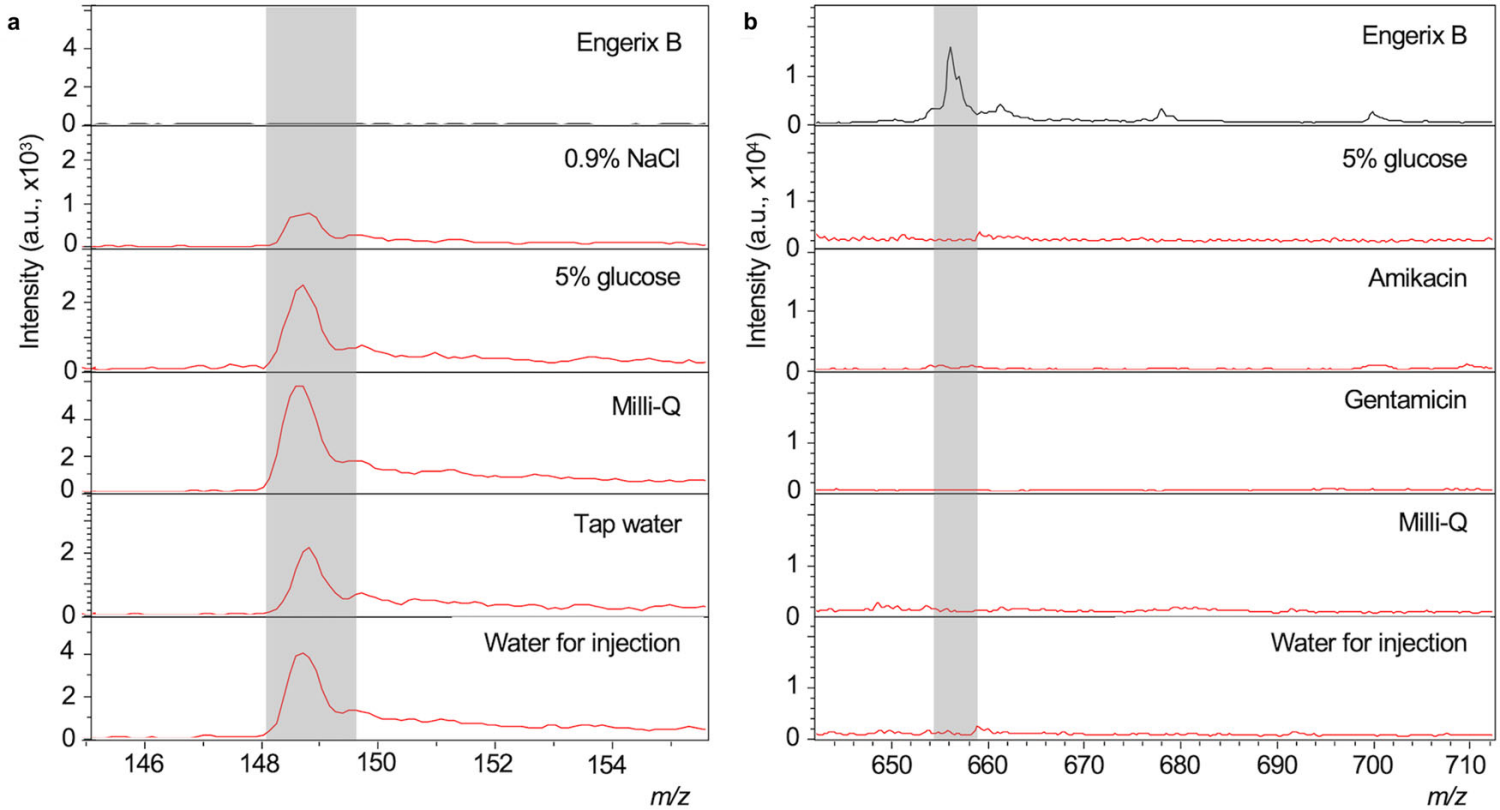
Exemplar peaks in raw spectra that could be targeted to confirm genuine Engerix B (Biotyper Sirius spectra). **a** Peaks present at *m/z* 148.661 in 0.9% (*m/V*) sodium chloride, 5% (*m/V*) glucose, tap water, Milli-Q and water for injection but not the genuine vaccine Engerix B. **b** A peak at *m/z* 656.246 unique to Engerix B against the falsified vaccine constituents 5% (*m/V*) glucose, Amikacin, Gentamicin, Milli-Q and water for injection.

Finally, we manually validated the multivariate model’s ability to predict important biomarker *m/z* values and identify candidate peaks. To do this, we interrogated the processed dataset independently from the PLS-DA model, comparing each individual *m/z* value’s peak intensity in the list of all identified peaks measured across all samples to look for statistically significant differences in mean abundance. For example, we compared each mass spectral peak from the Engerix B analysis with each peak from the analysis of the falsified vaccine constituents using ANOVA with the Dunnett multiple comparison test. In total 3699 *m/z* values were compared statistically, of these 143 showed statistically significant difference between Engerix B and at least one of the falsified vaccine constituents. Of the 143 significant peaks, 63 peaks were present in a falsified vaccine sample and not present at all in the genuine Engerix B, or vice versa. 63 peaks were, therefore, found to be unique differentiators of authenticity or falsification. It was, therefore, straightforward to unambiguously differentiate Engerix B from all other falsified vaccine surrogate samples using these peaks. The result of this analysis showed that there were many mass spectral peaks that could be used to discriminate the falsified from authentic vaccine samples. This provided strong redundancy and, therefore, demonstrated the potential for developing a database of distinguishing mass spectral peaks that could be used for vaccine authenticity testing. We have purposefully, on public health security grounds, not provided the full list of these features so as not to reveal specific features that may be used in any future databases for authenticity testing. However, Fig. 9 summarises the numbers of *m/z* features and those found to be significant and Fig. 10 presents two peaks from the group of 63 to illustrate. All of the Top 15 VIP *m/z* values from the PLS-DA modelling in Fig. 8a were also found in the 143 peaks identified by univariate statistical analysis for Engerix B, illustrating the overlap between the machine learning and manual inspection approaches for the identification of potential *“*biomarker*”* peaks suitable for differentiating genuine from fake vaccine samples.

## Discussion

Reports of substandard and falsified vaccines are increasing worldwide. In response, we have developed and validated a MALDI-MS sample analysis and data processing method and demonstrated its successful implementation in the context of vaccine authentication using four different authentic vaccines and known falsified vaccine surrogates. We chose two different MALDI systems that are distributed globally for the routine identification of pathological microorganisms in clinical laboratories. This pre-existing network of instrumentation, therefore, provides potential as a resource for future global supply chain monitoring. Combined with open-source machine learning and statistical analysis, we demonstrated our workflow could distinguish genuine from falsified vaccine surrogates accurately. To the best of our knowledge, this is the first time MALDI-MS has been used to successfully identify and discriminate vaccines and falsified surrogates using a machine-learning approach to data analysis.

A challenge in using MALDI-MS, compared to the other mass spectrometry platforms such as LC-MS and GC-MS, is its potential variability in the mass spectral peak intensities. We rigorously tested analytical, experimental and vaccine vial reproducibility and demonstrated that post-acquisition data processing was effective at minimising these effects. Our findings are commensurate with other studies in this regard; for example, in metabolomics applications where MALDI-MS has been applied successfully, in conjunction with machine learning, to identify metabolic differences in sera from lung cancer patients compared to healthy controls^53^. PLS-DA analysis demonstrated that a machine learning approach could be used to model MALDI-mass spectral peaks and their intensities for discriminating authentic and falsified vaccines. We also performed multivariate modelling on multiple authentic vaccines and in all cases, we were able to distinguish genuine from falsified vaccines using the validated PLS-DA model. In some cases, the different types of water and saline used in place of authentic vaccines were not fully resolved from each other, presumably due to their compositional similarity, but this did not detract from the PLS-DA model being able to reliably distinguish authentic vaccines from vaccine surrogates. The results of the PLS-DA modelling provided proof of principle that an unbiased, machine learning approach can successfully identify genuine vaccines from falsified constituents using MALDI-MS data and that this could be performed with very similar results using two different analytical instruments (Bruker Biotyper Sirius and bioMérieux VITEK MS) established and run at different laboratories by different people. Using univariate analysis, we also showed that 63 mass spectral peaks could be identified as uniquely present or absent in the Engerix B spectrum when compared to the falsified vaccine constituents. This illustrated strong potential for developing a database approach for vaccine authentication. The principle behind the identification of microorganisms with MALDI *'*Biotyping*'* instruments is the comparison of the mass spectrum of an unknown organism against a library of reference mass spectra^64^. Our results show this principle can also be applied to vaccine authentication given the large number of potentially diagnostic (discriminatory) peaks identified through data modelling. In summary, the benefit of MALDI analysis for vaccine authentication is two-fold: first, the method involves globally distributed MALDI technology, already deployed in a health context, making it potentially feasible to develop a global vaccine screening system. Second, using open-source machine learning with the full MALDI-mass spectrum would make it very difficult, if not impossible, to falsify vaccine surrogates that could pass through such a screening approach effectively. A careful assessment of how best to deploy the method in a real-world setting is required, and will be context-dependent. One approach could be to do so in combination with hand-held spectroscopic devices (e.g. as described in Mosca et al., 2023), deployed for rapid ‘on-site’ analysis. Suspicious samples could, in this way, be selected for confirmatory analysis using the MALDI-MS method developed here, potentially at a regional centre where MALDI-MS is already established for clinical testing applications.

The *m/z* values that proved most discriminatory in our study tended to be the compounds in the *m/z* 0–900 range and this demonstrated that diagnostic spectra were present for low-mass excipients of the vaccines themselves that we studied. This shows that selectivity is found across a wide range of adjuvants, the vaccine-specific profile of which would be more complex to falsify^65^. This molecular multiplexity can be seen as a benefit for vaccine authenticity testing as it does not rely on the presence or absence of a specific, or even a small number of, *'*biomarker*'* compounds that have the potential to be relatively easily introduced into falsified products. Whilst this study has focussed on developing a validated method and associated workflow using four genuine vaccines and eight vaccine surrogates known to have been used as falsified vaccines in real-world settings, we see no reasons why this approach could not be extended to other vaccines and liquid medicines such as insulin and biologics and associated falsified products.

This research demonstrates that a MALDI-MS method has the potential to be deployed in an international supply chain setting given that the instrumentation used is currently globally distributed for healthcare applications. The next steps the the porcess would be to develop and test a comprehensive online database for automated vaccine testing based on the methodology and workflow outlined here. Our research was aimed at the detection of vaccine falsification, however, evaluating the utility of MALDI-MS to detect a wider range of substandard vaccines, potentially brought about through inadvertent manufacturing errors or chemical degradation within supply chains (excursions in cold chain management, for example), would also be of interest in future work. We have provided a validated MALDI-MS method and proof of principle that it could be used in a range of vaccine quality control scenarios in the future.

## Methods

### Sample information

All samples were stored at 4 °C prior to analyses in accordance with manufacturers' storage recommendations and were in date (following labelled shelf-life) at the time of sample preparation and data acquisition. Table 3 provides details of the genuine vaccines used in this study, and the constituents that have been reported to be found in falsified vaccines, also tested in this study. Hyaluronic acid was obtained from Amazon (London, UK), Milli-Q water from a Milli-Q® Direct 8 water purification system (Merck Millipore, Darmstadt, Germany), and tap water from the Chemistry Research Laboratory, Oxford University. All other samples were procured through a local pharmacy in Oxford, UK.

**Table 3:** The four genuine vaccines and eight vaccine surrogates reported to be found in falsified vaccines used for analysis along with each manufacturer and batch number/part number

Vaccine/vaccine surrogate	Manufacturer	Batch/part number
Engerix B	GlaxoSmithKline	AHBVC999AL
Flucelvax Tetra	Seqirus	3079661A
Ixiaro	Valneva	JEV19F11A
Nimenrix	Pfizer	FE7696
0.9% *m/V* sodium chloride injection	Demo S.A Pharmaceutical Industry	24598/0002
5.0% *m/V* D-glucose	B/Braun	03551/0059
Amikacin 250 mg/mL	Hospira	05015997122159
Gentamicin 40 mg/mL	Demo S.A.	05208063001339
Hyaluronic acid	Guangzhou Ailian Cosmetic Co Ltd.	QB/T 2660
Tap water	Chemistry Research Laboratory, Oxford	N/A
Milli-Q water	Merck Millipore	N/A
Water for injection	Ph. Eur Demo S.A Pharmaceutical Industry	24598/001

*N/A* not applicable.

### Sample preparation

Samples were spotted onto MALDI target plates (Bruker, Billerica, MA, USA; part number (P/N) 1840375) and MS-DS target slides (bioMérieux, Basingstoke, UK), and prepared for analysis using an ASSIST PLUS pipetting robot equipped with an eight channel 12.5 μL VOYAGER adjustable tip spacing pipette and 12.5 μL GripTip pipette tips, all by INTEGRA Biosciences (Zizers, Switzerland; P/N 4505, 4721 and 6453 respectively). A dual reservoir adaptor fitted with a 25 mL divided reservoir (INTEGRA Biosciences; P/N 4547 and 4358 respectively) held the prepared α-cyano-4-hydroxycinnamic acid (HCCA/CHCA) matrix (bioMérieux CHCA matrix purchased from bioMérieux, (Basingstoke, UK; P/N 411071), Bruker standard solvent purchased from Sigma-Aldrich (Dorset, UK; P/N 900666), and Bruker portioned HCCA from Bruker (P/N 8255344)) in deck position A of the robot. Samples were pipetted manually into a 96-well plate (Sarstedt, Nümbrecht, Germany; P/N 72.1980.010) and placed in deck position B and the MALDI target plates were placed into a custom-built holder in position C. A pipetting programme was designed and uploaded to the VOYAGER pipette using the INTEGRA VIALAB software (version 2.1.1.0). For all sample preparations, the matrix and samples were mixed in a 1:1 (*V*/*V*) ratio and four replicates of 2 μL spots of the mixture were pipetted onto the MALDI target plates. The target plates were air-dried prior to MALDI-MS analysis. Although a pipetting robot was used for the preparation of samples, it should be noted that this is not mandatory and was used for efficiency rather than necessity.

### MALDI-MS data acquisition

Raw MS spectra were acquired via MALDI-mass spectrometry using a Bruker MALDI Biotyper Sirius (Bruker Daltonics, Bremen, Germany) and a bioMérieux VITEK MS (bioMérieux, Craponne, France). Each sample spot on the MALDI target plate was measured over three overlapping mass ranges: *m/z* 0–900, *m/z* 700–2,500 and *m/z* 2,000–20,000. Prior to sample analysis both MALDI-MS instruments were calibrated with Bruker antibiotic calibration standard (ACS), MBT Star-ACS, and Bruker bacterial test standard (BTS), both acquired from Bruker (product references 1818702 and 8255343, respectively).

For the Bruker MALDI Biotyper Sirius, custom AutoXecute methods were designed in Bruker flexControl software (version 3.4, Bruker Daltonics, Bremen, Germany) for the *'*MSP MALDI Biotarget 96 plate*'* geometry. Parameters for the three AutoXecute methods were as follows. Laser: MS/parent mode on and weight 2.00; initial laser power of 20% and maximal laser power set to 100%. Evaluation: *'*use masses from*'* was defined for each of the three specified mass ranges; *'*use background list' none; *'*ignore the 1 largest peak in the defined mass range*'* was not selected; MBT_Process processing method; smoothing and baseline subtraction off; peak resolution must be higher than 400; and digest/peptides with signal intensity *'*high*'*. Accumulation: MS/parent mode on; sum up 250 satisfactory shots in 50 shot steps; and dynamic termination off. Movement: random walk raster pattern with four shots at raster spot selected and quit sample after 60 subsequently failed judgments. Processing: flexAnalysis and Bio Tools MS methods set to none. Randomised acquisition sequences were generated for each plate of samples (using the 'RAND()' function in Microsoft Excel which generates random numbers), and implemented in the automatic run design within flexControl.

For the bioMérieux VITEK MS, data were acquired using the Shimadzu Biotech Launchpad software version 2.9.5.6 (Kratos Analytical, Manchester, UK). Parameters were as follows: laser power, 48; profiles, 100 per sample; shots, five accumulated per profile; maximum laser rep rate, 50.0. Pulsed extraction was optimised at 450 Da for *m/z* 0–900, 1600 Da for *m/z* 700–2500 and 13 kDa for *m/z* 2000–20,000. The regular circle bioMérieux CHCA raster was used with a diameter of 2 mm, 180 µm spacing and 109 points per target. Parent Data Export in the Method Editor was set as mzXML for the raw data file. SARAMIS Target Manager was used to create a list of samples with corresponding spot locations that was exported to Experiment Genie as a *.txt file. The *.txt file was opened in Microsoft Excel and the acquisition sequence was randomised. In auto experiment, the 4 × 48 Fleximass DS plate configuration was chosen and the *.txt file was set as a standard file in Import Experiment Genie before running the randomised acquisition sequence.

### Data processing

Spectra were exported from Bruker flexAnalysis (version 3.4, Bruker Daltonics) and Shimadzu Biotech Launchpad software (version 2.9.5.6). Raw spectra (.fid data files) from the Bruker Biotyper® Sirius were converted to .mzXML format with the CompassXport data export tool (Bruker Daltonics; version 4.0.0.8). The mzXML files from both Sirius and VITEK were imported into R studio and processed in R v4.1.2 using the MALDI Quant package. Baseline correction was performed using a *'*TopHat*'* algorithm and intensity calibration was performed with probabilistic quotient normalisation (PQN). Spectral alignment was performed using a half window size, signal-to-noise ratio (SNR) and tolerance of 7, 1 and 0.2, respectively. A locally weighted scatterplot smoothing (LOWESS) warping method was used. Peak detection used the same SNR, and half-window size parameters as previously defined and peak binning used a tolerance of 0.1. The resulting peak intensity matrices were exported as a .csv file for further analysis.

### Data analysis

Manual inspection of the raw mass spectra was performed by uploading the data files into Bruker flexAnalysis software (version 3.4) and Shimadzu Biotech Launchpad software (version 2.9.5.6) from the Sirius and VITEK instruments, respectively.

### Statistical analysis and data visualisation

Statistical analysis of the processed peak intensity matrices and visualisation of the data were performed using MetaboAnalyst (version 5.0, https://metaboanalyst.ca) and Workflow4metabolomics (https://workflow4metabolomics.org/). No data filtering was performed. Metaboanalyst was used to generate *'*heatmaps*'*, *'*hierarchical clustering dendrogram*'*, *'*principal component analysis (PCA)' and *'*partial least squares-discriminant analysis (PLS-DA)*'*. MetaboAnalyst data normalisation was performed by *'*sum' and Pareto scaled. Workflow4metabolomics was used for external validation of the multivariate models and the generation of confusion matrices. Two-way analysis of variance (ANOVA) with Dunnett multiple comparison test was performed in GraphPad Prism (GraphPad Software, Boston, MA, USA; version 9.4.1). Statistical analysis figures and graphical representations were created using both MetaboAnalyst and GraphPad Prism.

### Method validation

To ensure the MALDI-MS workflow was reproducible and reliable, having developed the method, both MALDI instruments were systematically validated for: (1) intra- and inter-day precision; repeatability and stability. Quality control (QC) samples were prepared as equimolar mixtures of all samples and spotted onto multiple positions on the MALDI plate in the same way as for experimental samples. With each spot representing a QC sample, 24 QC samples were each analysed on two different days, and the intra-day and inter-day precision was calculated as the percentage relative standard deviation (RSD) of the total ion count (TIC) across the mass range for each instrument. Intra-day reproducibility ranged from 28.75% to 41.96% and the combined inter-day precision was 34.85% and 39.89% for the Sirius and VITEK instruments, respectively. QC samples were measured under the same conditions for each instrument to estimate repeatability.

### Supplementary information

Supplementary Information

## Data Availability

The datasets from this study are available from the corresponding author on reasonable request.

## Code availability

The code used in this study is available from the corresponding author on reasonable request.

## References

[1] WHO. Substandard and falsified medical products. https://www.who.int/news-room/fact-sheets/detail/substandard-and-falsified-medical-products (2018).

[2] Yoshida, N. Research on the development of methods for detection of substandard and falsified medicines by clarifying their pharmaceutical characteristics using modern technology. *Biol. Pharm. Bull.* **47**, 878–885 (2024). 38692863 10.1248/bpb.b23-00749

[3] Medicine Quality Research Group University of Oxford. Medical product quality reports. https://www.iddo.org/mq/research/medical-product-quality-reports (2022).

[4] People may have got antibiotics at fake jab camp in Kolkata: cops. *Hindustan Times* (25 January 2021).

[5] Hashmi, F. Over 58,000 fake COVID-19 vaccine doses busted In China, 600 doses sent overseas, https://www.urdupoint.com/en/world/over-58000-fake-covid-19-vaccine-doses-buste-1164592.html (2021).

[6] Rajaram, P. Kolkata cops seize ‘dust and liquid’ vials from fake vaccination site where TMC MP Mimi Chakraborty took jab. *India Today* (25 June 2021).

[7] Fabi, R. & Costa, A. B. D. Indonesia begins re-vaccinating victims of fake drug ring. *Reuters* (18 July 2016).

[8] Rita, J. Locsin hints reported COVID-19 vaccine in Binondo could be fake, just ‘dextrose’. GMA News (21 December 2020).

[9] Hu, R. L., Fooks, A. R., Zhang, S. F., Liu, Y. & Zhang, F. Inferior rabies vaccine quality and low immunization coverage in dogs (Canis familiaris) in China. *Epidemiol. Infect.* **136**, 1556–1563 (2008). 18177524 10.1017/S0950268807000131 PMC2870756

[10] Offit, P. A. *The Cutter Incident: How America’s First Polio Vaccine Led to the Growing Vaccine Crisis* (Yale Univ. Press, 2007).

[11] Henson, K. E. R., Santiago, A. A. C. & Namqui, S. S. Counterfeit rabies vaccines: the Philippine experience. *Open Forum Infect. Dis.* **7**, ofaa313 (2020). 32855990 10.1093/ofid/ofaa313 PMC7443107

[12] WHO. Full list of WHO medical product alerts, https://www.who.int/teams/regulation-prequalification/incidents-and-SF/full-list-of-who-medical-product-alerts (2024) and https://www.nda.or.ug/wp-content/uploads/2022/02/Alert-1-2016_Fev_Falsified-AMARIL-yellow-fever-vaccine-SEARO_EN.pdf (2016).

[13] Nurlaela Arief, N., Karlinah, S., Setianti, Y. & Susilawati, S. Counterfeit vaccines in Indonesia: managing the issue through media. *J. Commun. Manag.* **22** (2018).

[14] WHO. WHO global surveillance and monitoring system for substandard and falsified medical products. https://apps.who.int/iris/handle/10665/326708 (2017).

[15] Mosca, S. et al. Innovative method for rapid detection of falsified COVID-19 vaccines through unopened vials using handheld spatially offset Raman spectroscopy (SORS). *Vaccine* **41**, 6960–6968 (2023). 37865599 10.1016/j.vaccine.2023.10.012 PMC7618537

[16] Pisani, E. et al. Substandard and falsified medicines: proposed methods for case finding and sentinel surveillance. *JMIR Public Health Surveill.* **7**, e29309 (2021). 34181563 10.2196/29309 PMC8406122

[17] Bharucha, T. et al. Repurposing rapid diagnostic tests to detect falsified vaccines in supply chains. *Vaccine* **42**, 1506–1511 (2024). 38355318 10.1016/j.vaccine.2024.01.019 PMC7618033

[18] Zuber, P. L. F. et al. Evolving pharmacovigilance requirements with novel vaccines and vaccine components. *BMJ Glob. Health* **6**, e003403 (2021). 10.1136/bmjgh-2020-003403 PMC8137242 34011500

[19] Caillet, C. et al. Evaluation of portable devices for medicine quality screening: lessons learnt, recommendations for implementation, and future priorities. *PLos Med.* **18**, e1003747 (2021). 34591861 10.1371/journal.pmed.1003747 PMC8483386

[20] McCullagh, J. S. O. & Oldham, N. J. *Mass Spectrometry* (Oxford Univ. Press, 2019).

[21] Blaise, B. J. et al. Statistical analysis in metabolic phenotyping. *Nat. Protoc.* **16**, 4299–4326 (2021). 34321638 10.1038/s41596-021-00579-1

[22] Galal, A., Talal, M. & Moustafa, A. Applications of machine learning in metabolomics: disease modeling and classification. *Front. Genet.* **13**, 1017340 (2022). 36506316 10.3389/fgene.2022.1017340 PMC9730048

[23] Pomyen, Y. et al. Deep metabolome: applications of deep learning in metabolomics. *Comput. Struct. Biotechnol. J.* **18**, 2818–2825 (2020). 33133423 10.1016/j.csbj.2020.09.033 PMC7575644

[24] Walsby-Tickle, J. et al. Anion-exchange chromatography mass spectrometry provides extensive coverage of primary metabolic pathways revealing altered metabolism in IDH1 mutant cells. *Commun. Biol.* **3**, 247 (2020). 32433536 10.1038/s42003-020-0957-6 PMC7239943

[25] Alldritt, I. et al. Metabolomics reveals diet-derived plant polyphenols accumulate in physiological bone. *Sci. Rep.* **9**, 8047 (2019). 31142795 10.1038/s41598-019-44390-1 PMC6541599

[26] Liebal, U. W., Phan, A. N. T., Sudhakar, M., Raman, K. & Blank, L. M. Machine learning applications for mass spectrometry-based metabolomics. *Metabolites* **10**, 243 (2020). 10.3390/metabo10060243 PMC7345470 32545768

[27] Neely, B. A. & Palmblad, M. Machine learning in proteomics and metabolomics. *J. Proteome Res.* **21**, 2553–2554 (2022). 36193949 10.1021/acs.jproteome.2c00566

[28] Feucherolles, M. et al. Combination of MALDI-TOF mass spectrometry and machine learning for rapid antimicrobial resistance screening: the case of Campylobacter spp. *Front. Microbiol.* **12**, 804484 (2021). 35250909 10.3389/fmicb.2021.804484 PMC8894766

[29] Lazari, L. C., Rosa-Fernandes, L. & Palmisano, G. Machine learning approaches to analyze MALDI-TOF mass spectrometry protein profiles. *Methods Mol. Biol.* **2511**, 375–394 (2022). 35838976 10.1007/978-1-0716-2395-4_29

[30] Rashidi, H. H. et al. Comparative performance of two automated machine learning platforms for COVID-19 detection by MALDI-TOF-MS. *PLos ONE* **17**, e0263954 (2022). 35905092 10.1371/journal.pone.0263954 PMC9337631

[31] Tran, N. K. et al. Novel application of automated machine learning with MALDI-TOF-MS for rapid high-throughput screening of COVID-19: a proof of concept. *Sci. Rep.* **11**, 8219 (2021). 33859233 10.1038/s41598-021-87463-w PMC8050054

[32] van Oosten, L. N. & Klein, C. D. Machine learning in mass spectrometry: a MALDI-TOF MS approach to phenotypic antibacterial screening. *J. Med. Chem.* **63**, 8849–8856 (2020). 32191034 10.1021/acs.jmedchem.0c00040

[33] Amini, A., Lodén, H. & Rundlöf, T. Identification of peptides and proteins in suspected illegal medicinal products using MALDI-TOF-MS. *Eur. Pharm. Rev.* **24** (2019).

[34] Bronzel, J. L. Jr, Milagre, C. D. F. & Milagre, H. M. S. Analysis of low molecular weight compounds using MALDI- and LDI-TOF-MS: direct detection of active pharmaceutical ingredients in different formulations. *J. Mass Spectrom.* **52**, 752–758 (2017). 28806859 10.1002/jms.3984

[35] Debeljak, Ž. et al. MALDI TOF mass spectrometry imaging of blood smear: method development and evaluation. *Int. J. Mol. Sci.* **22**, 585 (2021). 10.3390/ijms22020585 PMC7827909 33430160

[36] Di Francesco, L. et al. A MALDI-TOF MS approach for mammalian, human, and formula milks’ profiling. *Nutrients* **10**, 1238 (2018). 10.3390/nu10091238 PMC6163840 30189627

[37] Hortin, G. L. The MALDI-TOF mass spectrometric view of the plasma proteome and peptidome. *Clin. Chem.* **52**, 1223–1237 (2006). 16644871 10.1373/clinchem.2006.069252

[38] Kober, S. L., Hollert, H. & Frohme, M. Quantification of nitroaromatic explosives in contaminated soil using MALDI-TOF mass spectrometry. *Anal. Bioanal. Chem.* **411**, 5993–6003 (2019). 31278552 10.1007/s00216-019-01976-y PMC6706601

[39] Siricord, C. & O’Brien, P. A. MALDI-TOF mass spectrometry can be used for detection of pathogenic microorganisms in soil. *Australas. Plant Path* **37**, 543–545 (2008).

[40] Lévesque, S. et al. A side by side comparison of Bruker Biotyper and VITEK MS: utility of MALDI-TOF MS technology for microorganism identification in a public health reference laboratory. *PLos ONE* **10**, e0144878 (2015). 26658918 10.1371/journal.pone.0144878 PMC4689555

[41] Pollard, A. J. & Bijker, E. M. A guide to vaccinology: from basic principles to new developments. *Nat. Rev. Immunol.* **21**, 83–100 (2021). 33353987 10.1038/s41577-020-00479-7 PMC7754704

[42] Gibb, S. & Strimmer, K. MALDIquant: a versatile R package for the analysis of mass spectrometry data. *Bioinformatics* **28**, 2270–2271 (2012). 22796955 10.1093/bioinformatics/bts447

[43] Pang, Z. et al. MetaboAnalyst 5.0: narrowing the gap between raw spectra and functional insights. *Nucleic Acids Res.* **49**, W388–W396 (2021). 34019663 10.1093/nar/gkab382 PMC8265181

[44] Engerix B 20 micrograms/1 ml Suspension for injection in pre-filled syringe. https://www.medicines.org.uk/emc/product/1637/smpc#gref (2023).

[45] Flucelvax Tetra. https://www.ema.europa.eu/en/medicines/human/EPAR/flucelvax-tetra (2023).

[46] Ixiaro. https://www.ema.europa.eu/en/medicines/human/EPAR/ixiaro (2023).

[47] Nimenrix. https://www.ema.europa.eu/en/medicines/human/EPAR/nimenrix (2024).

[48] Costa, A. B. D. & Kapoor, K. Indonesian lawmakers seek seizure of unapproved vaccines amid fake drug scare. https://www.reuters.com/article/us-indonesia-health-crime-idUKKCN0ZD1LB (2016).

[49] Fałszywe szczepionki w Polsce. “To powszechnie dostępny środek”. https://www.polsatnews.pl/wiadomosc/2021-04-22/falszywe-szczepionki-w-polsce-to-powszechnie-dostepny-srodek/ (22 April 2021).

[50] Topić Popović, N., Kazazić, S. P., Bojanić, K., Strunjak-Perović, I. & Čož-Rakovac, R. Sample preparation and culture condition effects on MALDI-TOF MS identification of bacteria: a review. *Mass Spectrom. Rev.* **42**, 1589–1603 (2023). 34642960 10.1002/mas.21739

[51] Oberle, M. et al. The technical and biological reproducibility of matrix-assisted laser desorption ionization-time of flight mass spectrometry (MALDI-TOF MS) based typing: employment of bioinformatics in a multicenter study. *PLos ONE* **11**, e0164260 (2016). 27798637 10.1371/journal.pone.0164260 PMC5087883

[52] Albrethsen, J. Reproducibility in protein profiling by MALDI-TOF mass spectrometry. *Clin. Chem.* **53**, 852–858 (2007). 17395711 10.1373/clinchem.2006.082644

[53] Lai, X. et al. Combining MALDI-MS with machine learning for metabolomic characterization of lung cancer patient sera. *Anal. Methods* **14**, 499–507 (2022). 34981796 10.1039/D1AY01940F

[54] Ryan, C. G., Clayton, E., Griffin, W. L., Sie, S. H. & Cousens, D. R. SNIP, a statistics-sensitive background treatment for the quantitative analysis of PIXE spectra in geoscience applications. *Nucl. Instrum. Methods Phys. Res. Sect. B Beam Interact. Mater. At.* **34**, 396–402 (1988). 10.1016/0168-583X(88)90063-8

[55] Sternberg, S. R. Grayscale morphology. *Comput. Vis. Graph. Image Process.* **35**, 333–355 (1986). 10.1016/0734-189X(86)90004-6

[56] Dieterle, F., Ross, A., Schlotterbeck, G. & Senn, H. Probabilistic quotient normalization as robust method to account for dilution of complex biological mixtures. Application in 1H NMR metabonomics. *Anal. Chem.* **78**, 4281–4290 (2006). 16808434 10.1021/ac051632c

[57] He, Q. P., Wang, J., Mobley, J. A., Richman, J. & Grizzle, W. E. Self-calibrated warping for mass spectra alignment. *Cancer Inf.* **10**, 65–82 (2011). 10.4137/CIN.S6358 PMC3085421 21552490

[58] Wehrens, R., Bloemberg, T. G. & Eilers, P. H. Fast parametric time warping of peak lists. *Bioinformatics* **31**, 3063–3065 (2015). 25971741 10.1093/bioinformatics/btv299

[59] Stephen, C. G. & Dane, A. H. in Metab*olo*mics *-* Fund*amentals and Applications* (ed. Prasain, J.) Ch. 4 (IntechOpen, 2016).

[60] Worley, B. & Powers, R. Multivariate analysis in metabolomics. *Curr. Metabolomics* **1**, 92–107 (2013). 26078916 10.2174/2213235X11301010092 PMC4465187

[61] Ralbovsky, N. M. & Smith, J. P. Machine learning for prediction, classification, and identification of immobilized enzymes for biocatalysis. *Pharm. Res.* **40**, 1479–1490 (2023). 36653518 10.1007/s11095-022-03457-x

[62] Xia, J. & Wishart, D. S. Using MetaboAnalyst 3.0 for comprehensive metabolomics data analysis. *Curr. Protoc. Bioinformatics* **55**, 14.10.11–14.10.91 (2016). 10.1002/cpbi.11 27603023

[63] Thevenot, E. A., Roux, A., Xu, Y., Ezan, E. & Junot, C. Analysis of the human adult urinary metabolome variations with age, body mass index and gender by implementing a comprehensive workflow for univariate and OPLS statistical analyses. *J. Proteome Res.* **14**, 3322–3335 (2015). 26088811 10.1021/acs.jproteome.5b00354

[64] Asare, P. T. et al. A MALDI-TOF MS library for rapid identification of human commensal gut bacteria from the class Clostridia. *Front. Microbiol.* **14**, 1104707 (2023). 36896425 10.3389/fmicb.2023.1104707 PMC9990839

[65] Jain, S., Venkataraman, A., Wechsler, M. E. & Peppas, N. A. Messenger RNA-based vaccines: past, present, and future directions in the context of the COVID-19 pandemic. *Adv. Drug Deliv. Rev.* **179**, 114000 (2021). 34637846 10.1016/j.addr.2021.114000 PMC8502079

