# Supplementary material for: Using matrix assisted laser desorption ionisation mass spectrometry combined with machine learning for vaccine authenticity screening
Source: NPJ Vaccines. 2024 Aug 28;9:155. doi: 10.1038/s41541-024-00946-5 (PMC11358428; doi:10.1038/s41541-024-00946-5)
Supplement: Supplementary file 1 — Supplementary Information [file 41541_2024_946_MOESM1_ESM.pdf]

Supplementary Information

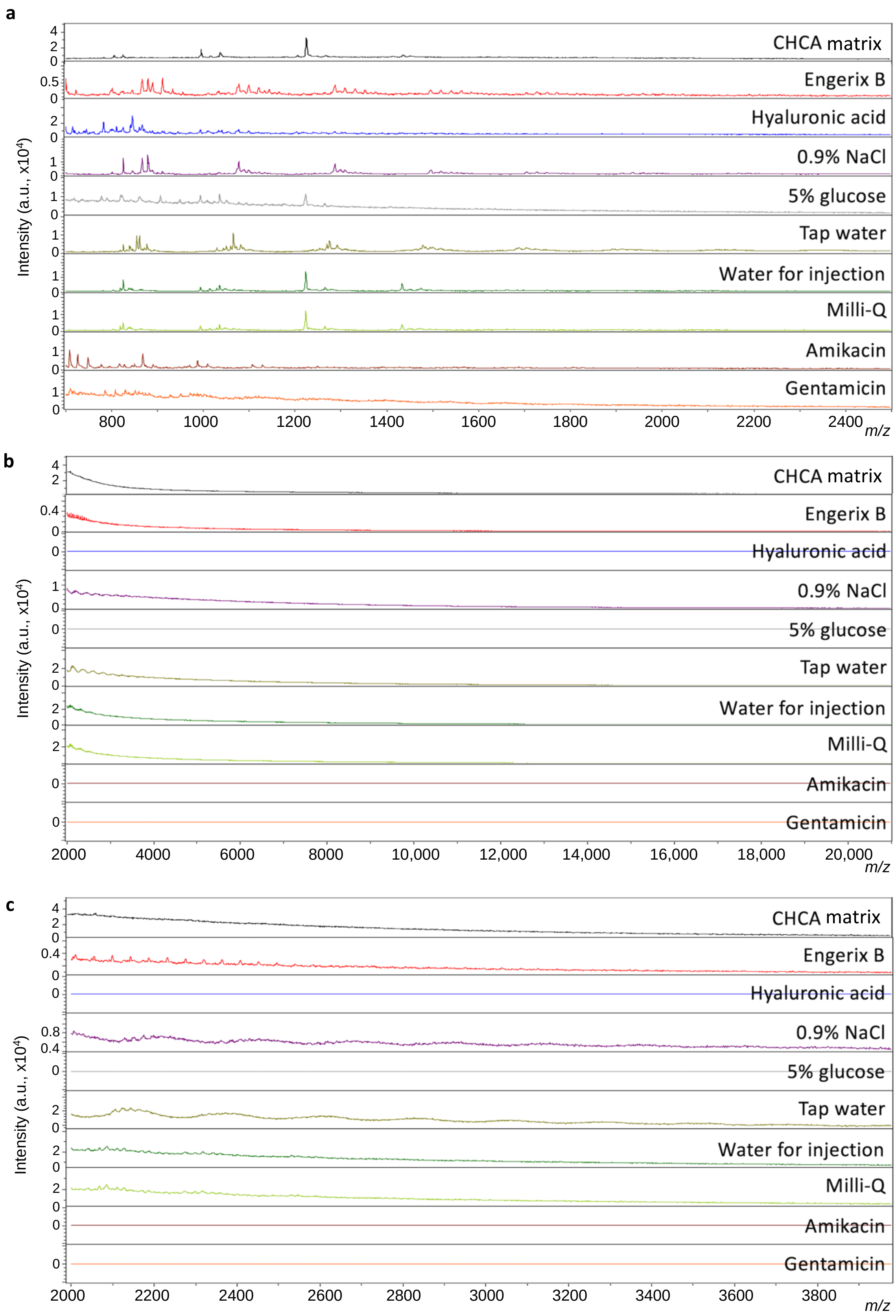

Supplementary Figure 1. Bruker Sirius representative mass spectra for α-cyano-4-hydroxycinnamic acid (CHCA) matrix, Engerix B vaccine and 8 samples of other compounds and mixtures previously reported as being constituents of or could be

used as 'falsified vaccine' products. (a) 700–2500 *m/z* mass range. (b) 2000–20,000 *m*/z mass range. (c) zoomed high mass range 2000–4000 *m/z*. Where baseline spectra are present, the instrument failed to detect peaks for those samples at the given mass range.

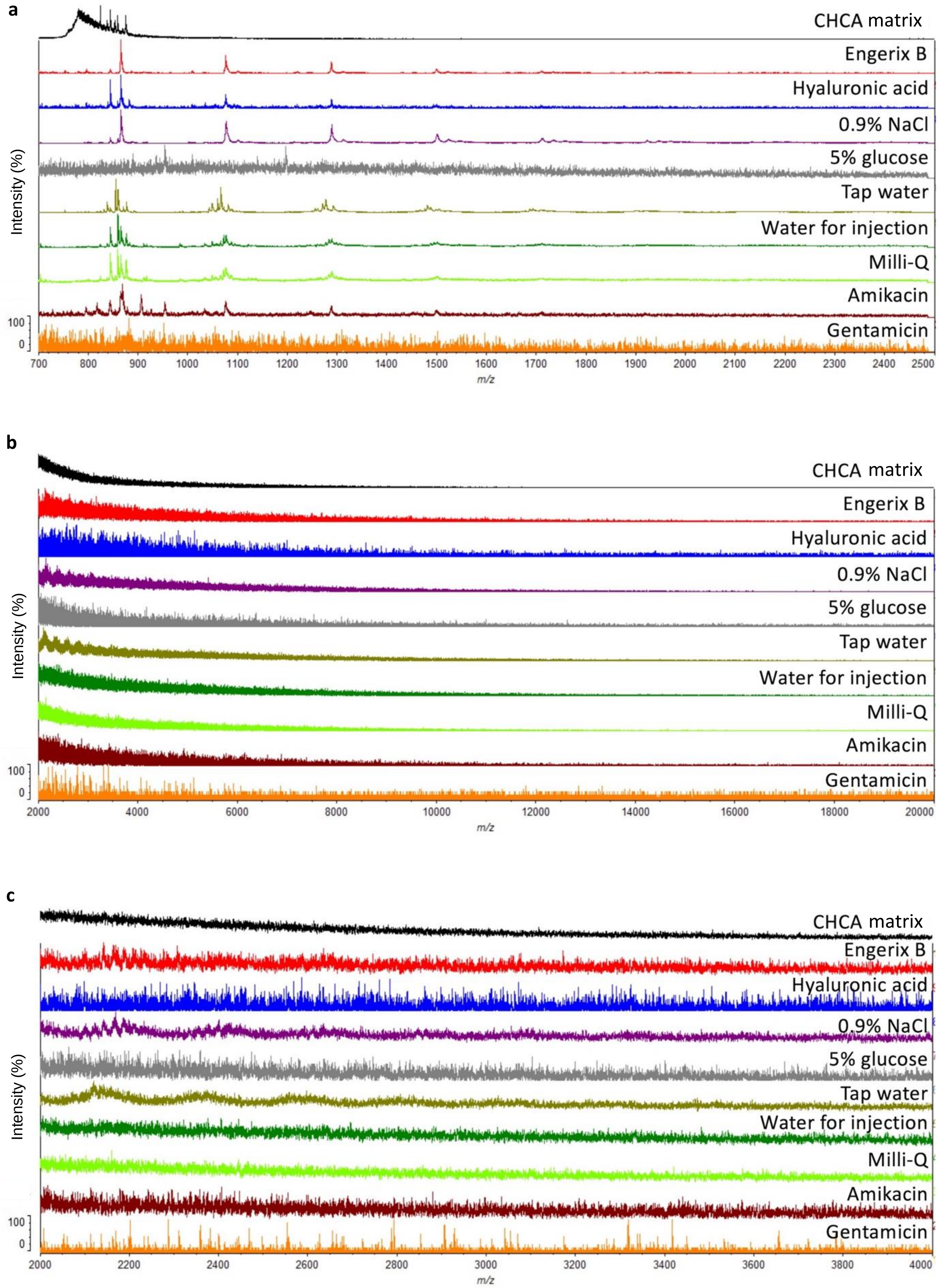

Supplementary Figure 2. VITEK MS representative mass spectra for α-cyano-4-hydroxycinnamic acid (CHCA) matrix, Engerix B vaccine and 8 samples of other compounds and mixtures previously reported as being constituents of or could be used as

‘falsified vaccine’ products. (a) 700–2500 *m/z* mass range. (b) 2000–20,000 *m/z* mass range. (c) zoomed high mass range 2000–4000 *m/z*.

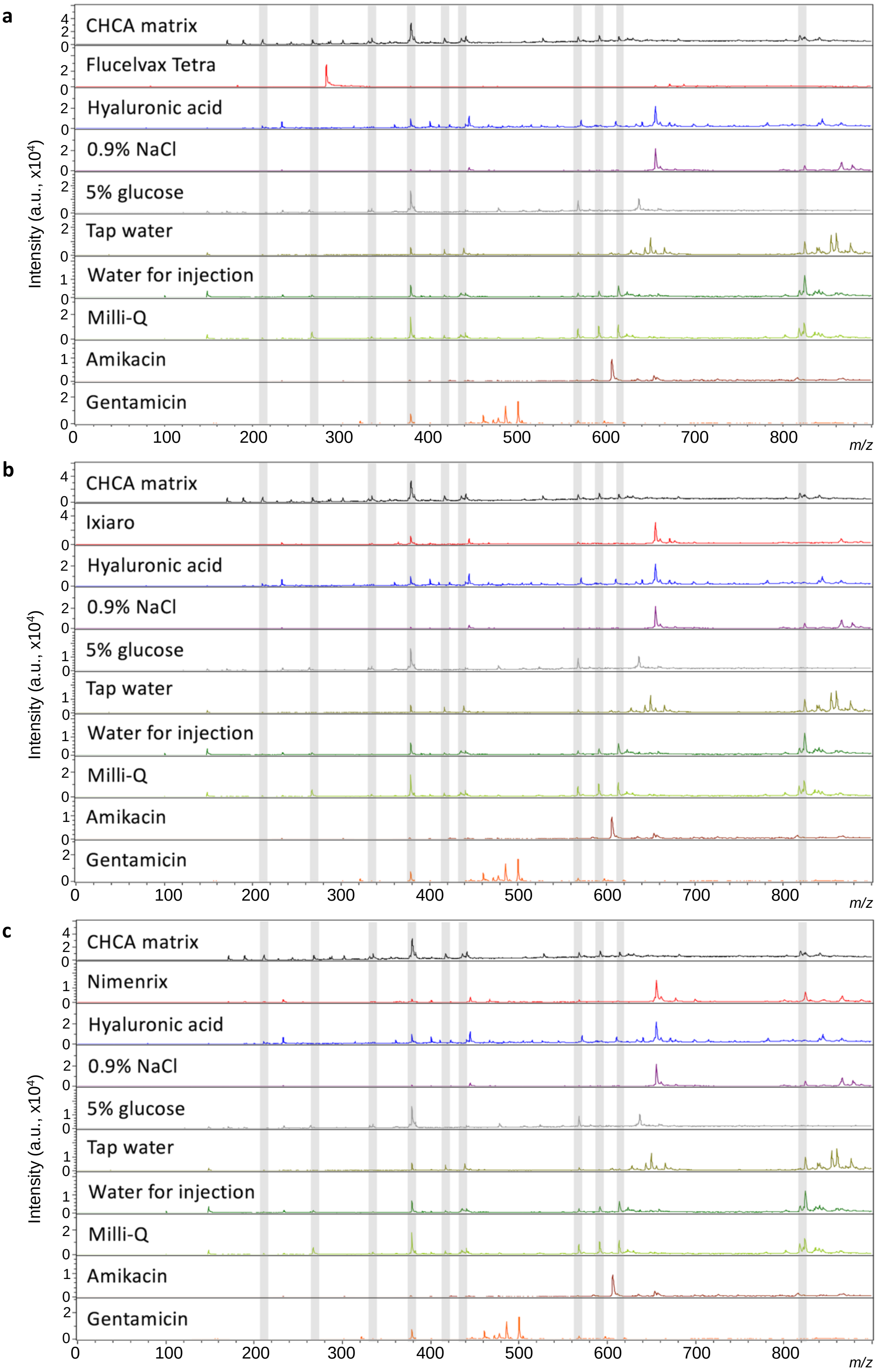

Supplementary Figure 3. Biotyper Sirius representative mass spectra (*m/z* 0–900) for α-cyano-4-hydroxycinnamic acid (CHCA) matrix, genuine vaccine and 8 samples of other compounds and mixtures previously reported as being constituents of or could be used as ‘falsified vaccine’ products. (a) Ixiaro vaccine. (b) Flucelvax Tetra vaccine. (c) Nimenrix vaccine. Where matrix peaks are present in the sample spectra, these are indicated by the shaded bars. Through the presence, absence, and

relative intensity ratios of peaks in the spectra, each genuine vaccine can be distinguished from the falsified constituents by manual inspection.

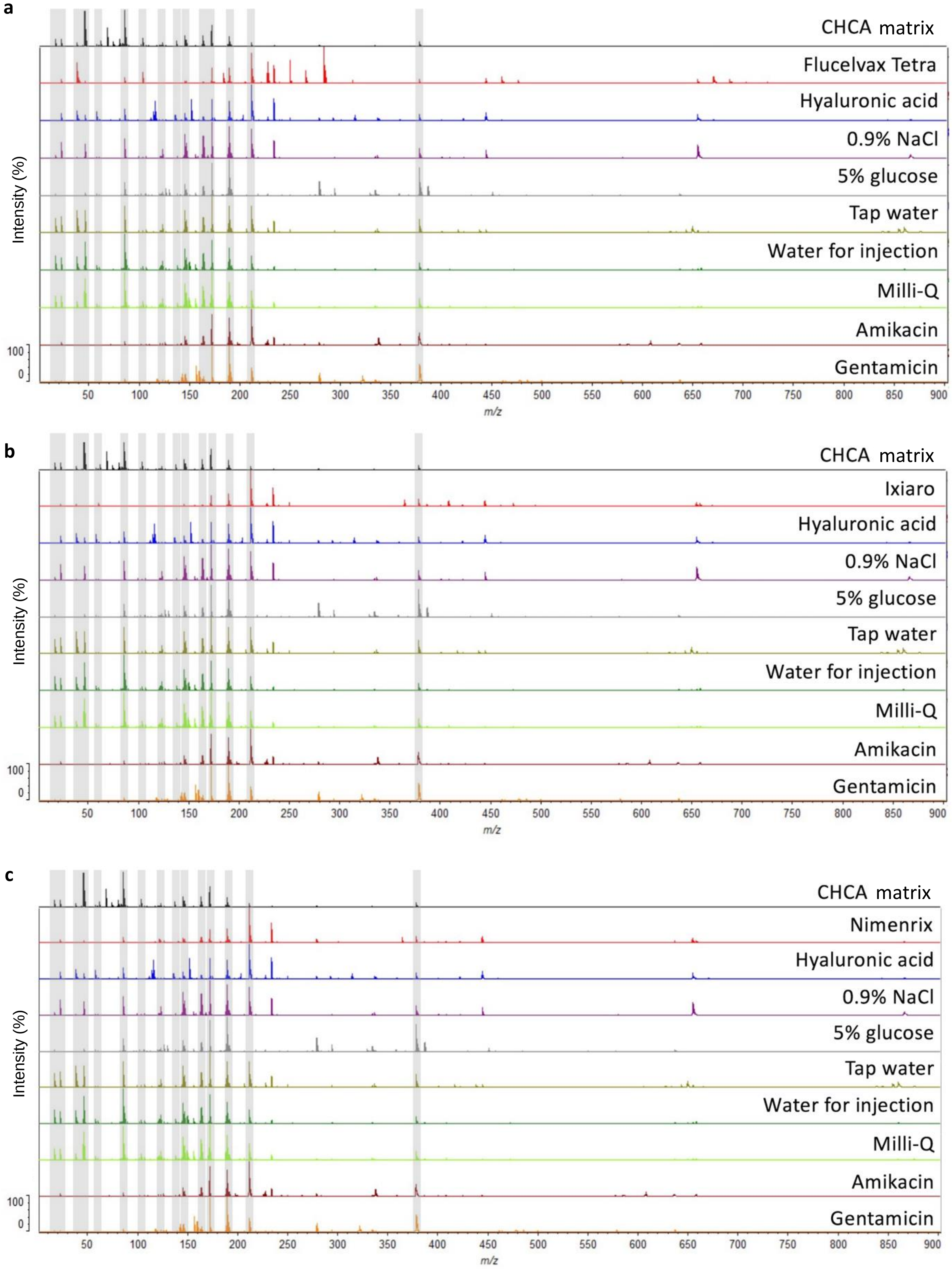

Supplementary Figure 4. VITEK MS representative mass spectra (*m/z* 0–900) for α-cyano-4-hydroxycinnamic acid (CHCA) matrix, genuine vaccine and 8 samples of other compounds and mixtures previously reported as being constituents of or

could be used as 'falsified vaccine' products. (a) Ixiaro vaccine. (b) Flucelvax Tetra vaccine. (c) Nimenrix vaccine. Where matrix peaks are present in the sample spectra these are indicated by the shaded bars. Through the presence, absence, and relative intensity ratios of peaks in the spectra, each genuine vaccine can be distinguished from the falsified constituents by manual inspection.

Sample	RSD (%)
0.9% NaCl	40.53
5% glucose	64.28
Amikacin	52.42
Gentamicin	36.49
Hyaluronic acid	28.75
Tap water	18.71
Milli-Q water	9.61
Water for injection	24.22
Engerix B	15.90
Flucelvax Tetra	24.34
Ixiaro	49.29
Nimenrix	94.84
Quality control	32.32

Supplementary Table 1. Evaluated reproducibility of the raw data from the VITEK MS (0–900 *m/z*). The percentage relative standard deviation (RSD) values calculated from the total ion intensities of all 12 sample replicates and 24 quality control replicates are given.

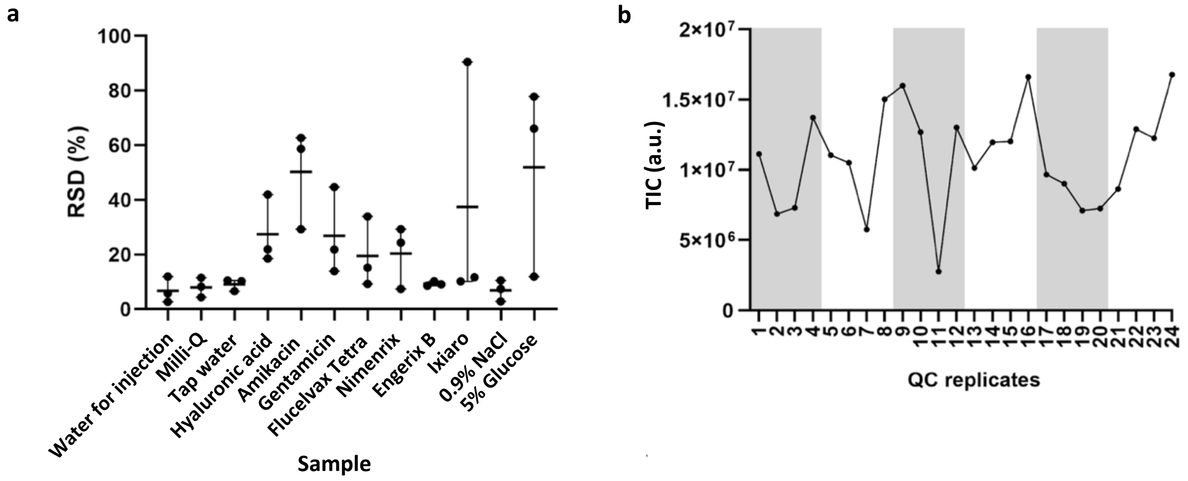

Supplementary Figure 5. Analytical reproducibility for each sample was measured using consecutive replicate analyses. VITEK MS data is used for analysis here. (a) The percentage relative standard deviation (RSD) values for each vial per sample are plotted showing the range and mean. (b) The total ion count (TIC) for each quality control (QC) sample replicate plotted in consecutive run order shows no particular bias (replicates spotted on different MALDI plates are alternately shaded/white).

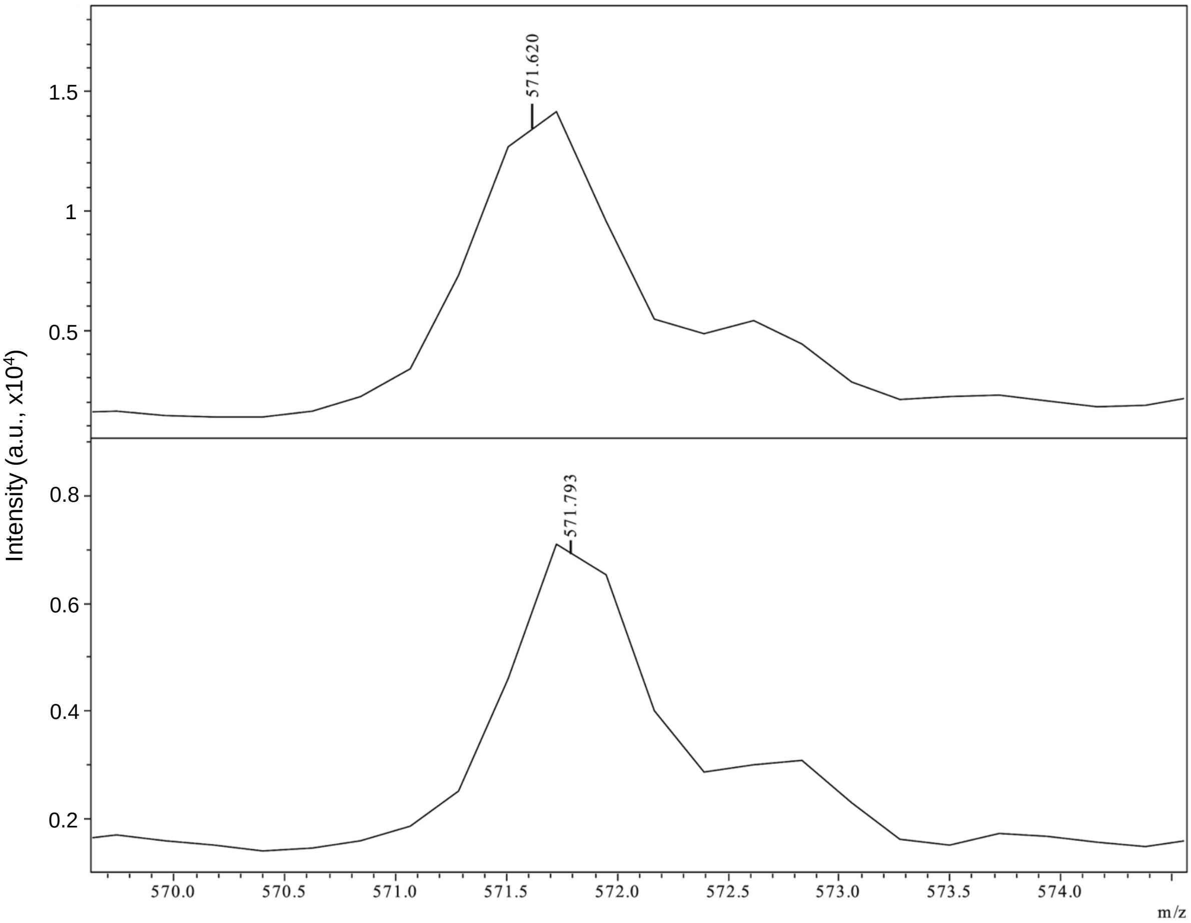

Supplementary Figure 6. Two mass spectra replicates of the same sample showing the centroided *m/*z values for a peak at approximately 572 *m/z*. The differences in peak shape that can occur between spectra, such as the different peak apex shapes presented here, can result in different *m/z* values being picked for the same peak during processing.

m/z	0.9% NaCl	0.9% NaCl	5% glucose	5% glucose	Amikacin	Amikacin	Gentamicin	Gentamicin
586.643	NA	NA	NA	NA	NA	NA	NA	NA
586.867	NA	NA	NA	NA	NA	NA	NA	NA
587.091	NA	NA	NA	NA	NA	NA	NA	NA
587.315	NA	NA	NA	NA	NA	NA	NA	NA
587.540	NA	NA	NA	NA	NA	NA	NA	NA
587.764	NA	NA	NA	NA	NA	NA	NA	NA
587.988	NA	NA	NA	NA	NA	NA	0.00584	0.00653
588.213	NA	NA	0.00213	0.00171	NA	NA	NA	NA
588.437	NA	NA	NA	NA	NA	NA	NA	NA
588.661	NA	NA	NA	NA	NA	NA	NA	NA
588.886	NA	NA	NA	NA	NA	NA	NA	NA
589.110	NA	NA	NA	NA	NA	NA	NA	NA
589.335	NA	NA	NA	NA	NA	NA	NA	NA
589.560	NA	NA	NA	NA	NA	NA	NA	NA
589.784	NA	NA	NA	NA	NA	NA	NA	NA
590.009	0.00263	0.00213	NA	NA	NA	NA	0.00238	0.00389
590.234	NA	NA	NA	NA	NA	NA	NA	NA
590.458	NA	NA	NA	NA	NA	NA	NA	NA
590.683	NA	NA	NA	NA	NA	NA	NA	NA
592.015	NA	NA	0.00296	0.00324	0.00218	0.00159	0.00219	0.00290
593.384	NA	NA	NA	NA	NA	NA	NA	NA
593.610	NA	NA	NA	NA	NA	NA	NA	NA
593.835	0.0016	0.00102	NA	NA	NA	NA	NA	NA
594.061	NA	NA	NA	NA	NA	NA	NA	NA
594.286	NA	NA	NA	NA	NA	NA	NA	NA
594.512	NA	NA	NA	NA	NA	NA	NA	NA
594.737	NA	NA	NA	NA	NA	NA	NA	NA

NA: no peak intensity detected.

Supplementary Table 2. Example of data table of results for statistical analysis produced by MALDIquant workflow (Biotyper Sirius data). Column 1 contains the *m/z* values from peak picking using the MALDIquant workflow. The probabilistic quotient normalised intensities for each *m/z* for 2 replicates are shown for 0.9% NaCl, 5% glucose, amikacin, and gentamicin. The table covers the mass range 586.643–594.737 *m/z*.

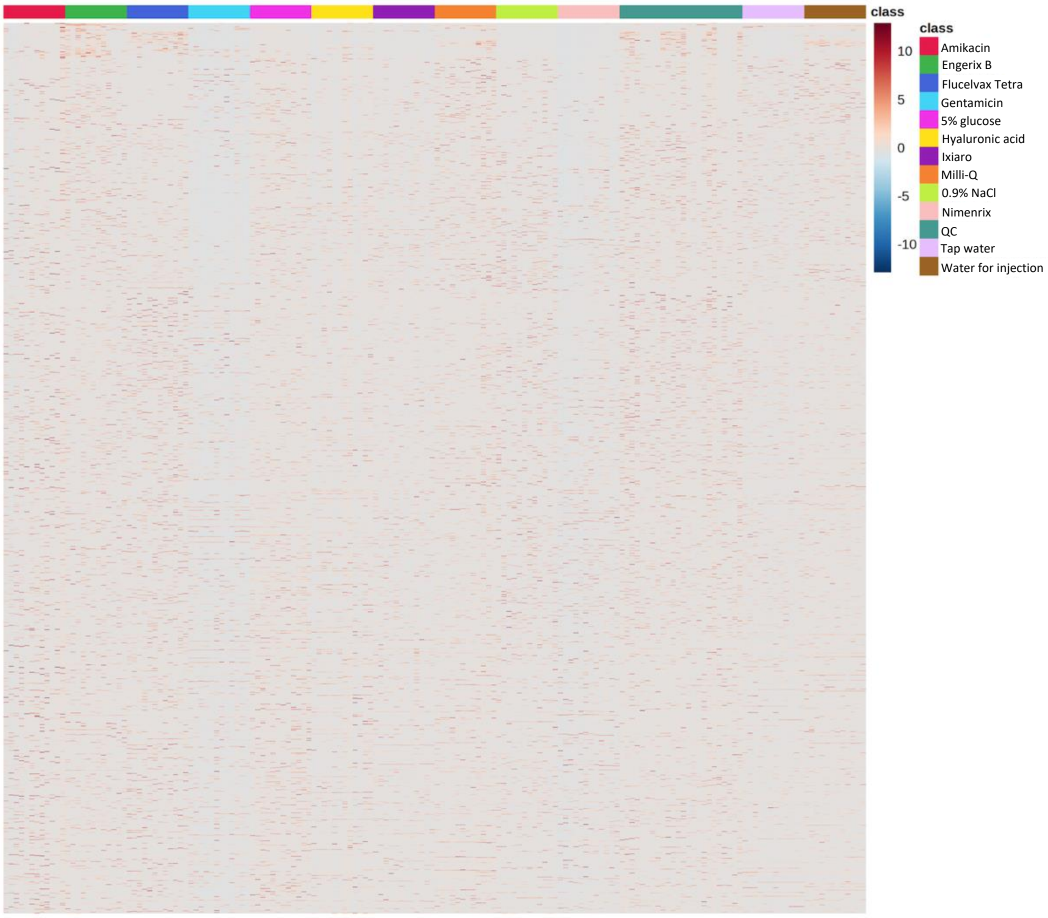

Supplementary Figure 7. Heatmap of all compound-feature intensities across all samples shows no individual samples of class outliers after data processing (Bruker Sirius data). The colour groups represent the 12 analytical replicates for each sample (columns). Each row represents one of the 3699 *m/z* features produced via MALDIquant data processing and is colour coded red through blue to show relative intensity for the feature for each sample. A stronger red colour corresponds to higher relative intensity, whereas stronger blue corresponds to lower relative intensity. QC: quality control.

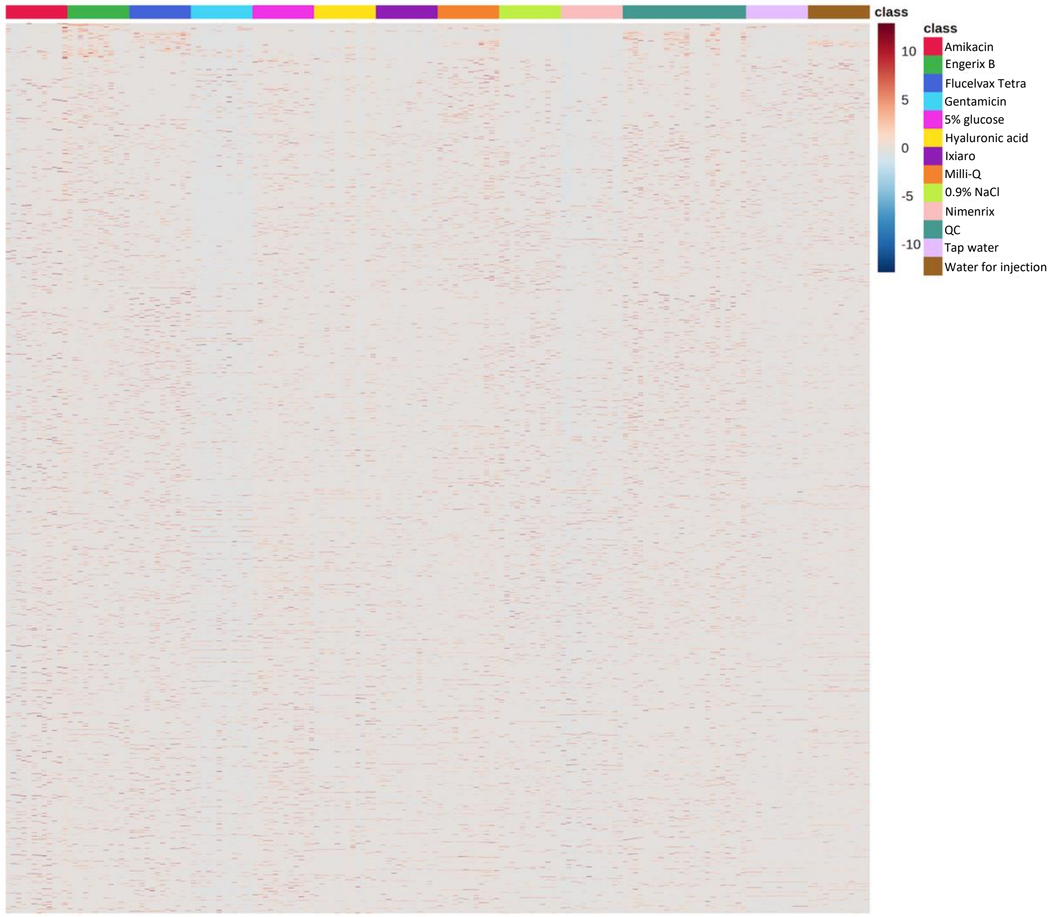

Supplementary Figure 8. Heatmap of all compound-feature intensities across all samples shows no individual samples of class outliers after data processing (VITEK MS data). The colour groups represent the 12 analytical replicates for each sample (columns). Each row represents one of the 2817 *m/z* features produced via MALDIquant data processing and is colour coded red through blue to show relative intensity for the feature for each sample. A stronger red colour corresponds to higher relative intensity, whereas stronger blue corresponds to lower relative intensity. QC: quality control.

Measure	1 component	2 components	3 components	4 components	5 components	6 components	7 components	8 components
Accuracy	0.21422	0.36038	0.50824	0.59574	0.77108	0.86831	0.92724	0.98221
R2	0.92383	0.97765	0.98709	0.99433	0.99716	0.99861	0.99939	0.99969
Q2	0.83353	0.89795	0.90239	0.90766	0.90694	0.9057	0.9045	0.90402

Supplementary Table 3. Cross validation for partial least squares discriminant analysis of Engerix B compared to falsified vaccine surrogates (Bruker Sirius, 0–900 *m/*z). The numerical values for the performance of accuracy, R-squared (R2), and Q-squared (Q2) are displayed for each number of components used.

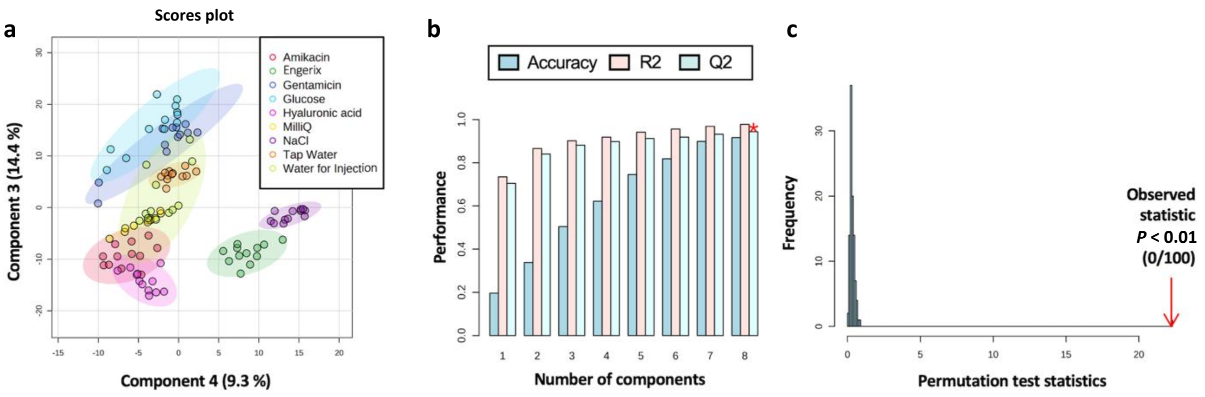

d

Measure	1 component	2 components	3 components	4 components	5 components	6 components	7 components	8 components
Accuracy	0.19593	0.33759	0.50391	0.62154	0.74542	0.81798	0.89925	0.91613
R2	0.73441	0.86553	0.90175	0.91863	0.94198	0.95583	0.96912	0.97796
Q2	0.70408	0.84078	0.88159	0.89829	0.91249	0.91813	0.93179	0.94327

Supplementary Figure 9. VITEK MS partial least-squares discriminant analysis (PLS-DA) of authentic vaccine Engerix B and all falsified vaccine constituents, 0–900 *m/z*. (a) PLS-DA two-dimensional scores plot shows samples group clustering. The most discrimination between Engerix B and the falsified constituents was seen between principal components 3 and 4 which is shown here. (b) Cross validation shows a minimum of 8 components (mass spectral peaks) are required to differentiate the experimental groups for the best Q-squared (Q2) value (shown by *). The Performance axis indicates the predictive ability of the model. (c) Permutation testing showed the model was significant with p-value <0.01. (d) R-squared (R2), Q2 and accuracy values for the cross-validation in B.

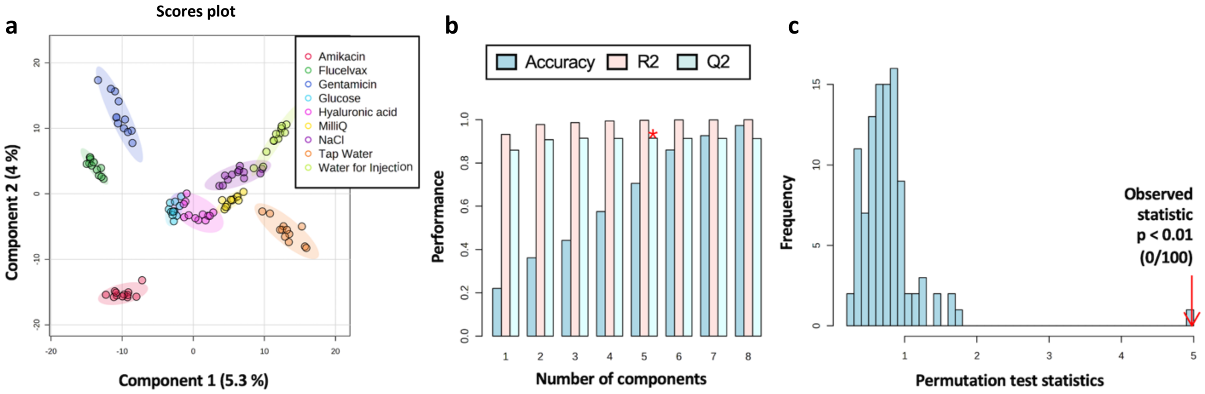

d

Measure	1 component	2 components	3 components	4 components	5 components	6 components	7 components	8 components
Accuracy	0.21975	0.36118	0.44146	0.57541	0.70501	0.86035	0.92563	0.97238
R2	0.93212	0.97752	0.98669	0.99436	0.99684	0.99856	0.99941	0.99971
Q2	0.85902	0.90799	0.91436	0.91382	0.91475	0.91383	0.91374	0.91264

Supplementary Figure 10. Biotyper Sirius partial least-squares discriminant analysis (PLS-DA) of authentic vaccine Flucelvax Tetra and all falsified vaccine constituents. (a) PLS-DA two-dimensional scores plot shows samples group clustering. (b) Cross validation shows a minimum of 5 components (mass spectral peaks) are required to differentiate the experimental groups for the best Q-squared (Q2) value (shown by *). The Performance axis indicates the predictive ability of the model. (c) Permutation testing showed the model was significant with $P < 0.01$. (d) R-squared (R2), Q2 and accuracy values for the cross-validation in B.

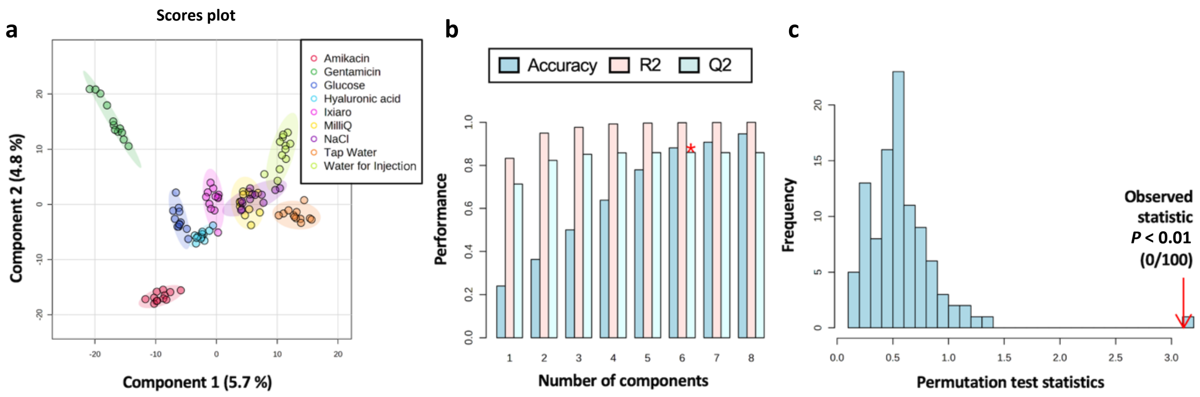

d

Measure	1 component	2 components	3 components	4 components	5 components	6 components	7 components	8 components
Accuracy	0.23979	0.36295	0.50002	0.63871	0.77925	0.88103	0.90751	0.94621
R2	0.83308	0.94922	0.97687	0.99201	0.99631	0.99836	0.99913	0.99963
Q2	0.71354	0.82345	0.85089	0.85836	0.85882	0.86029	0.85859	0.85917

Supplementary Figure 11. Biotyper Sirius partial least-squares discriminant analysis (PLS-DA) of authentic vaccine Ixiaro and all falsified vaccine constituents. (a) PLS-DA two-dimensional scores plot shows samples group clustering. (b) Cross validation shows a minimum of 6 components (mass spectral peaks) are required to differentiate the experimental groups for the best Q-squared (Q2) value (shown by *). The Performance axis indicates the predictive ability of the model. (c) Permutation testing showed the model was significant *with P* < 0.01. (d) R-squared (R2), Q2 and accuracy values for the cross-validation in B.

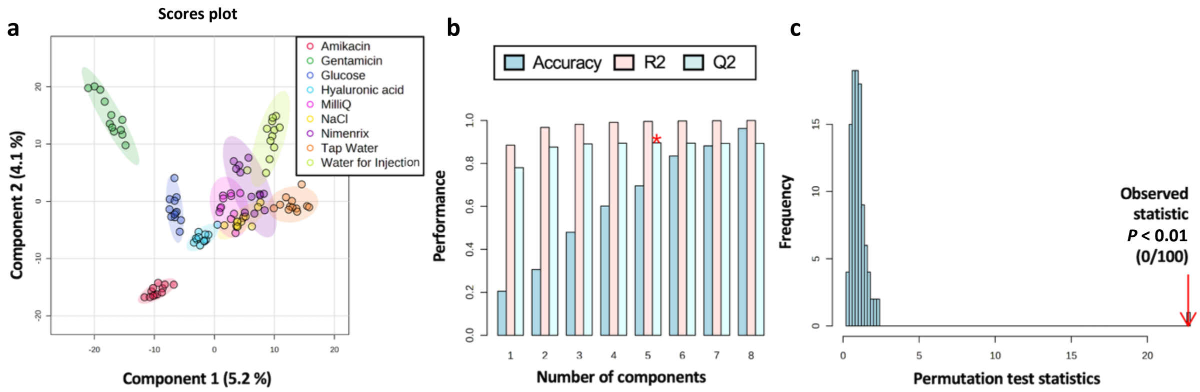

d

Measure	1 component	2 components	3 components	4 components	5 components	6 components	7 components	8 components
Accuracy	0.2043	0.30619	0.48002	0.60148	0.69562	0.83483	0.8821	0.96312
R2	0.88508	0.9675	0.9823	0.99153	0.99599	0.99835	0.99927	0.99966
Q2	0.78019	0.87646	0.89077	0.89394	0.89576	0.89426	0.89334	0.89279

Supplementary Figure 12. Biotyper Sirius partial least-squares discriminant analysis (PLS-DA) of authentic vaccine Nimenrix and all falsified vaccine constituents. (a) PLS-DA two-dimensional scores plot shows samples group clustering. (b) Cross validation shows a minimum of 5 components (mass spectral peaks) are required to differentiate the experimental groups for the best Q-squared (Q2) value (shown by *). The Performance axis indicates the predictive ability of the model. (c) Permutation testing showed the model was significant *with P* < 0.01. (d) R-squared (R2), Q2 and accuracy values for the cross-validation in B.

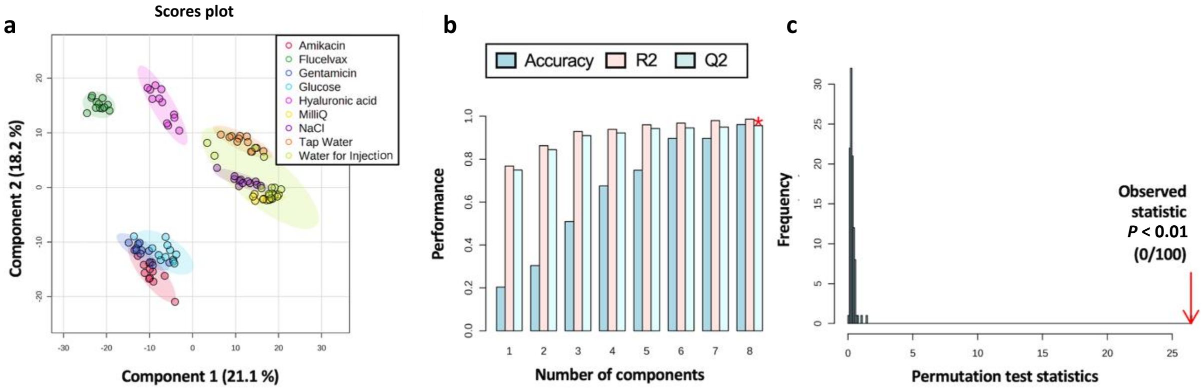

d

Measure	1 component	2 components	3 components	4 components	5 components	6 components	7 components	8 components
Accuracy	0.20381	0.304	0.50876	0.67505	0.7481	0.89686	0.89686	0.96143
R2	0.7679	0.86241	0.92888	0.93894	0.96031	0.96804	0.97982	0.98656
Q2	0.74868	0.84442	0.90948	0.92188	0.94219	0.94596	0.94957	0.95604

Supplementary Figure 13. VITEK MS partial least-squares discriminant analysis (PLS-DA) of authentic vaccine Flucelvax Tetra and all falsified vaccine constituents. (a) PLS-DA two-dimensional scores plot shows samples group clustering. (b) Cross validation shows a minimum of 8 components (mass spectral peaks) are required to differentiate the experimental groups for the best Q-squared (Q2) value (shown by *). The Performance axis indicates the predictive ability of the model. (c) Permutation testing showed the model was significant *with P* < 0.01. (d) R-squared (R2), Q2 and accuracy values for the cross-validation in B

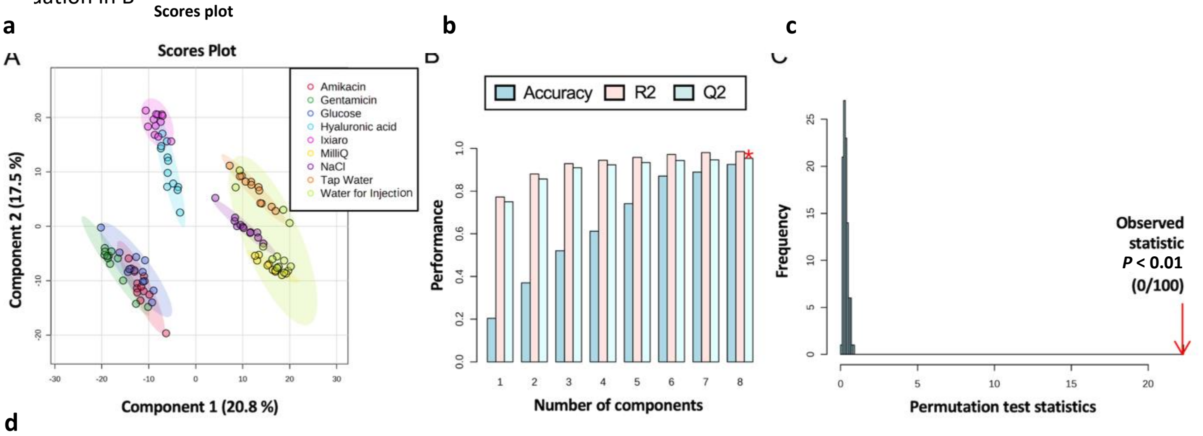

d

Measure	1 component	2 components	3 components	4 components	5 components	6 components	7 components	8 components
Accuracy	0.20365	0.37	0.52025	0.61214	0.74133	0.87088	0.88906	0.9255
R2	0.77264	0.88014	0.9292	0.9443	0.95805	0.97216	0.98059	0.98564
Q2	0.75012	0.85731	0.9092	0.92357	0.93425	0.94381	0.94676	0.95285

Supplementary Figure 14. VITEK MS partial least-squares discriminant analysis (PLS-DA) of authentic vaccine Ixiaro and all falsified vaccine constituents. (a) PLS-DA two-dimensional scores plot shows samples group clustering. (b) Cross validation shows a minimum of 8 components (mass spectral peaks) are required to differentiate the experimental groups for the best Q-squared (Q2) value (shown by *). The Performance axis indicates the predictive ability of the model. (c) Permutation testing showed the model was significant *with P* < 0.01. (d) R-squared (R2), Q2 and accuracy values for the cross-validation in B.

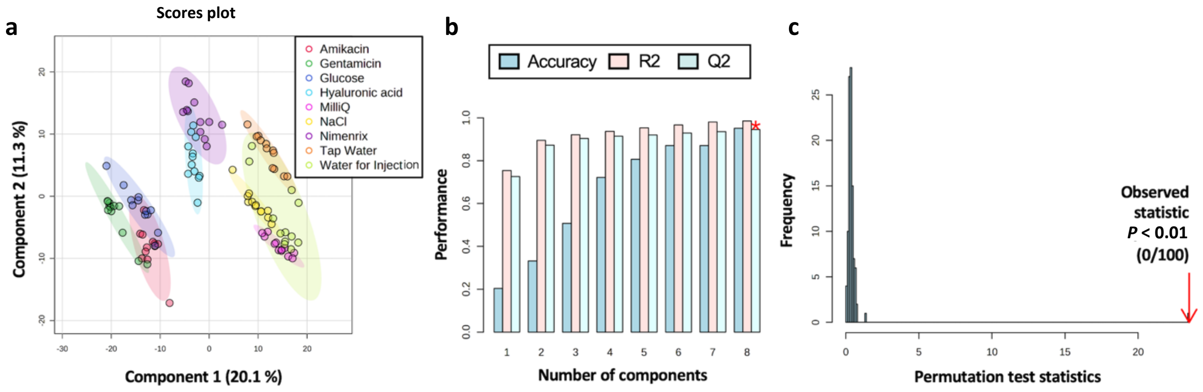

d

Measure	1 component	2 components	3 components	4 components	5 components	6 components	7 components	8 components
Accuracy	0.20369	0.3321	0.5074	0.72186	0.80684	0.87083	0.87083	0.95124
R2	0.75418	0.89504	0.92108	0.93646	0.95299	0.96698	0.98058	0.98501
Q2	0.72615	0.87293	0.90355	0.91466	0.92027	0.92862	0.93566	0.94542

Supplementary Figure 15. VITEK MS partial least-squares discriminant analysis (PLS-DA) of authentic vaccine Nimenrix and all falsified vaccine constituents. (a) PLS-DA two-dimensional scores plot shows samples group clustering. (b) Cross validation shows a minimum of 8 components (mass spectral peaks) are required to differentiate the experimental groups for the best Q-squared (Q2) value (shown by *). The Performance axis indicates the predictive ability of the model. (c) Permutation testing showed the model was significant *with P* < 0.01. (d) R-squared (R2), Q2 and accuracy values for the cross-validation in B.

Falsified constituents and genuine vaccine	Amikacin	Engerix B	Gentamicin	Glucose	Hyaluronic acid	MilliQ	NaCl	Tap water	Water for injection
Amikacin	12	0	0	0	0	0	0	0	0
Engerix B	0	12	0	0	0	0	0	0	0
Gentamicin	0	0	12	0	0	0	0	0	0
Glucose	0	0	0	12	0	0	0	0	0
Hyaluronic acid	0	0	0	0	12	0	0	0	0
MilliQ	0	0	0	0	0	12	0	0	0
NaCl	0	0	0	0	0	0	12	0	0
Tap water	0	0	0	0	0	0	12	0	0
Water for injection	0	0	0	0	0	12	0	0	0

Supplementary Table 4. Engerix B confusion matrix using all-data (Sirius MS).

Falsified constituents and genuine vaccine	Amikacin	Engerix B	Gentamicin	Glucose	Hyaluronic acid	MilliQ	NaCl	Tap water	Water for injection
Amikacin	10	0	0	0	0	0	0	0	0
Engerix B	0	10	0	0	0	0	0	0	0
Gentamicin	0	0	10	0	0	0	0	0	0
Glucose	0	0	0	9	0	1	0	0	0
Hyaluronic acid	0	0	0	0	10	0	0	0	0
MilliQ	0	0	0	0	0	10	0	0	0
NaCl	0	0	0	0	0	0	10	0	0
Tap water	0	0	0	0	0	0	0	10	0
Water for injection	0	0	0	0	0	2	0	0	8

Supplementary Table 5. Engerix B confusion matrix using the training-set data (Sirius MS).

Falsified constituents and genuine vaccine	Amikacin	Engerix B	Gentamicin	Glucose	Hyaluronic acid	MilliQ	NaCl	Tap water	Water for injection
Amikacin	2	0	0	0	0	0	0	0	0
Engerix B	0	2	0	0	0	0	0	0	0
Gentamicin	0	0	2	0	0	0	0	0	0
Glucose	0	0	0	2	0	0	0	0	0
Hyaluronic acid	0	0	0	0	2	0	0	0	0
MilliQ	0	0	0	0	0	2	0	0	0
NaCl	0	0	0	0	0	0	2	0	0
Tap water	0	0	0	0	0	0	0	2	0
Water for injection	0	0	0	0	0	2	0	0	0

Supplementary Table 6. Engerix B confusion matrix using the test-set data (Sirius MS).

Falsified constituents and genuine vaccine	Amikacin	Flucelvax	Gentamicin	Glucose	Hyaluronic acid	MilliQ	NaCl	Tap water	Water for injection
Amikacin	12	0	0	0	0	0	0	0	0
Flucelvax	0	12	0	0	0	0	0	0	0
Gentamicin	0	0	12	0	0	0	0	0	0
Glucose	1	0	0	11	0	0	0	0	0
Hyaluronic acid	0	0	0	0	12	0	0	0	0
MilliQ	0	0	0	0	0	0	0	0	12
NaCl	0	0	0	0	0	0	12	0	0
Tap water	0	0	0	0	0	0	0	12	0
Water for injection	0	0	0	0	0	0	0	0	12

Supplementary Table 7. Flucelvax confusion matrix using all-data (Sirius MS).

Falsified constituents and genuine vaccine	Amikacin	Flucelvax	Gentamicin	Glucose	Hyaluronic acid	MilliQ	NaCl	Tap water	Water for injection
Amikacin	10	0	0	0	0	0	0	0	0
Flucelvax	0	10	0	0	0	0	0	0	0
Gentamicin	0	0	10	0	0	0	0	0	0
Glucose	2	0	0	7	0	1	0	0	0
Hyaluronic acid	0	0	0	0	10	0	0	0	0
MilliQ	0	0	0	0	0	10	0	0	0
NaCl	0	0	0	0	0	0	10	0	0
Tap water	0	0	0	0	0	0	0	10	0
Water for injection	0	0	0	0	0	7	0	0	3

Supplementary Table 8. Flucelvax confusion matrix using the training-set data (Sirius MS).

Falsified constituents and genuine vaccine	Amikacin	Flucelvax	Gentamicin	Glucose	Hyaluronic acid	MilliQ	NaCl	Tap water	Water for injection
Amikacin	2	0	0	0	0	0	0	0	0
Flucelvax	0	2	0	0	0	0	0	0	0
Gentamicin	0	0	2	0	0	0	0	0	0
Glucose	0	0	0	2	0	0	0	0	0
Hyaluronic acid	0	0	0	0	2	0	0	0	0
MilliQ	0	0	0	0	0	2	0	0	0
NaCl	0	0	0	0	0	0	2	0	0
Tap water	0	0	0	0	0	0	0	2	0
Water for injection	0	0	0	0	0	2	0	0	0

Supplementary Table 9. Flucelvax confusion matrix using the test-set data (Sirius MS).

Falsified constituents and genuine vaccine	Amikacin	Gentamicin	Glucose	Hyaluronic acid	MilliQ	NaCl	Nimenrix	Tap water	Water for injection
Amikacin	12	0	0	0	0	0	0	0	0
Gentamicin	0	12	0	0	0	0	0	0	0
Glucose	0	0	12	0	0	0	0	0	0
Hyaluronic acid	0	0	0	12	0	0	0	0	0
MilliQ	0	0	0	0	0	0	0	0	12
NaCl	0	0	0	0	0	12	0	0	0
Nimenrix	0	0	0	0	0	0	12	0	0
Tap water	0	0	0	0	0	0	0	12	0
Water for injection	0	0	0	0	0	0	0	0	12

Supplementary Table 10. Nimenrix confusion matrix using the all-data (Sirius MS).

Falsified constituents and genuine vaccine	Amikacin	Gentamicin	Glucose	Hyaluronic acid	MilliQ	NaCl	Nimenrix	Tap water	Water for injection
Amikacin	10	0	0	0	0	0	0	0	0
Gentamicin	0	10	0	0	0	0	0	0	0
Glucose	0	0	10	0	0	0	0	0	0
Hyaluronic acid	0	0	0	10	0	0	0	0	0
MilliQ	0	0	0	0	9	0	0	0	1
NaCl	0	0	0	0	0	10	0	0	0
Nimenrix	0	0	0	0	0	0	10	0	0
Tap water	0	0	0	0	0	0	0	10	0
Water for injection	0	0	0	0	5	0	0	0	5

Supplementary Table 11. Nimenrix confusion matrix using the training-set data (Sirius MS).

Falsified constituents and genuine vaccine	Amikacin	Gentamicin	Glucose	Hyaluronic acid	MilliQ	NaCl	Nimenrix	Tap water	Water for injection
Amikacin	2	0	0	0	0	0	0	0	0
Gentamicin	0	2	0	0	0	0	0	0	0
Glucose	0	0	2	0	0	0	0	0	0
Hyaluronic acid	0	0	0	2	0	0	0	0	0
MilliQ	0	0	0	0	2	0	0	0	0
NaCl	0	0	0	0	0	2	0	0	0
Nimenrix	0	0	0	0	0	0	2	0	0
Tap water	0	0	0	0	0	0	0	2	0
Water for injection	0	0	0	0	2	0	0	0	0

Supplementary Table 12. Nimenrix confusion matrix using the test-set data (Sirius MS).

Falsified constituents and genuine vaccine	Amikacin	Gentamicin	Glucose	Hyaluronic acid	Ixiaro	MilliQ	NaCl	Tap water	Water for injection
Amikacin	12	0	0	0	0	0	0	0	0
Gentamicin	0	12	0	0	0	0	0	0	0
Glucose	0	0	12	0	0	0	0	0	0
Hyaluronic acid	0	0	0	12	0	0	0	0	0
Ixiaro	0	0	0	0	12	0	0	0	0
MilliQ	0	0	0	0	0	0	0	0	12
NaCl	0	0	0	0	0	0	12	0	0
Tap water	0	0	0	0	0	0	0	12	0
Water for injection	0	0	0	0	0	0	0	0	12

Supplementary Table 13. Ixiaro confusion matrix using the test-set data (Sirius MS).

Falsified constituents and genuine vaccine	Amikacin	Gentamicin	Glucose	Hyaluronic acid	Ixiaro	MilliQ	NaCl	Tap water	Water for injection
Amikacin	10	0	0	0	0	0	0	0	0
Gentamicin	0	10	0	0	0	0	0	0	0
Glucose	0	0	10	0	0	0	0	0	0
Hyaluronic acid	0	0	0	10	0	0	0	0	0
Ixiaro	0	0	0	0	10	0	0	0	0
MilliQ	0	0	0	0	0	10	0	0	0
NaCl	0	0	0	0	0	0	10	0	0
Tap water	0	0	0	0	0	0	0	10	0
Water for injection	0	0	0	0	0	10	0	0	0

Supplementary Table 14. Ixiaro confusion matrix using the training-set data (Sirius MS).

Falsified constituents and genuine vaccine	Amikacin	Gentamicin	Glucose	Hyaluronic acid	Ixiaro	MilliQ	NaCl	Tap water	Water for injection
Amikacin	2	0	0	0	0	0	0	0	0
Gentamicin	0	2	0	0	0	0	0	0	0
Glucose	0	0	2	0	0	0	0	0	0
Hyaluronic acid	0	0	0	2	0	0	0	0	0
Ixiaro	0	0	0	0	2	0	0	0	0
MilliQ	0	0	0	0	0	2	0	0	0
NaCl	0	0	0	0	0	0	2	0	0
Tap water	0	0	0	0	0	0	0	2	0
Water for injection	0	0	0	0	0	2	0	0	0

Supplementary Table 15. Ixiaro confusion matrix using the test-set data (Sirius MS).

Falsified constituents and genuine vaccine	Amikacin	Engerix B	Gentamicin	Glucose	Hyaluronic acid	MilliQ	NaCl	Tap water	Water for injection
Amikacin	12	0	0	0	0	0	0	0	0
Engerix	0	12	0	0	0	0	0	0	0
Gentamicin	0	0	12	0	0	0	0	0	0
Glucose	0	0	0	12	0	0	0	0	0
Hyaluronic Acid	0	0	0	0	12	0	0	0	0
MilliQ	0	0	0	0	0	12	0	0	0
NaCl	0	5	0	0	0	0	7	0	0
Tap water	0	0	0	0	0	0	0	12	0
Water for injection	0	0	0	0	0	8	0	1	3

Supplementary Table 16. Engerix B confusion matrix using the all-data (VITEK MS).

Falsified constituents and geniune vaccine	Amikacin	Engerix B	Gentamicin	Glucose	Hyaluronic acid	MilliQ	NaCl	Tap water	Water for injection
Amikacin	10	0	0	0	0	0	0	0	0
Engerix	0	10	0	0	0	0	0	0	0
Gentamicin	0	0	10	0	0	0	0	0	0
Glucose	0	0	0	10	0	0	0	0	0
Hyaluronic Acid	0	0	0	0	10	0	0	0	0
MilliQ	0	0	0	0	0	0	0	0	10
NaCl	0	4	0	0	0	0	6	0	0
Tap water	0	0	0	0	0	0	0	10	0
Water for injection	0	0	0	0	0	0	0	0	10

Supplementary Table 17. Engerix B confusion matrix using the training-set data (VITEK MS).

Falsified constituents and geniune vaccine	Amikacin	Engerix B	Gentamicin	Glucose	Hyaluronic acid	MilliQ	NaCl	Tap water	Water for injection
Amikacin	2	0	0	0	0	0	0	0	0
Engerix	0	2	0	0	0	0	0	0	0
Gentamicin	0	0	2	0	0	0	0	0	0
Glucose	0	0	0	2	0	0	0	0	0
Hyaluronic Acid	0	0	0	0	2	0	0	0	0
MilliQ	0	0	0	0	0	0	0	0	2
NaCl	0	1	0	0	0	0	1	0	0
Tap water	0	0	0	0	0	0	0	2	0
Water for injection	0	0	0	0	0	0	0	2	0

Supplementary Table 18. Engerix B confusion matrix using the test-set data (VITEK MS).

Falsified constituents and genuine vaccine	Amikacin	Flucelvax	Gentamicin	Glucose	Hyaluronic acid	MilliQ	NaCl	Tap water	Water for injection
Amikacin	12	0	0	0	0	0	0	0	0
Flucelvax	0	12	0	0	0	0	0	0	0
Gentamicin	0	0	12	0	0	0	0	0	0
Glucose	0	0	0	12	0	0	0	0	0
Hyaluronic Acid	0	0	0	0	12	0	0	0	0
MilliQ	0	0	0	0	0	12	0	0	0
NaCl	0	0	0	0	0	0	12	0	0
Tap water	0	0	0	0	0	0	0	12	0
Water for injection	0	1	0	0	0	8	0	0	3

Supplementary Table 19. Flucelvax confusion matrix using the all-data (VITEK MS).

Falsified constituents and genuine vaccine	Amikacin	Flucelvax	Gentamicin	Glucose	Hyaluronic acid	MilliQ	NaCl	Tap water	Water for injection
Amikacin	10	0	0	0	0	0	0	0	0
Flucelvax	0	10	0	0	0	0	0	0	0
Gentamicin	0	0	10	0	0	0	0	0	0
Glucose	0	0	0	10	0	0	0	0	0
Hyaluronic Acid	0	0	0	0	10	0	0	0	0
MilliQ	0	0	0	0	0	0	0	0	10
NaCl	0	0	0	0	0	0	10	0	0
Tap water	0	0	0	0	0	0	0	10	0
Water for injection	0	0	0	0	0	0	0	0	10

Supplementary Table 20. Flucelvax confusion matrix using the training-data (VITEK MS).

Falsified constituents and genuine vaccine	Amikacin	Flucelvax	Gentamicin	Glucose	Hyaluronic acid	MilliQ	NaCl	Tap water	Water for injection
Amikacin	2	0	0	0	0	0	0	0	0
Flucelvax	0	2	0	0	0	0	0	0	0
Gentamicin	0	0	2	0	0	0	0	0	0
Glucose	0	0	0	2	0	0	0	0	0
Hyaluronic Acid	0	0	0	0	2	0	0	0	0
MilliQ	0	0	0	0	0	0	0	0	2
NaCl	0	0	0	0	0	0	2	0	0
Tap water	0	0	0	0	0	0	0	2	0
Water for injection	0	1	0	0	0	0	0	0	1

Supplementary Table 21. Flucelvax confusion matrix using the test-set data (VITEK MS).

Falsified constituents and genuine vaccine	Amikacin	Gentamicin	Glucose	Hyaluronic acid	MilliQ	NaCl	Nimenrix	Tap water	Water for injection
Amikacin	12	0	0	0	0	0	0	0	0
Gentamicin	0	12	0	0	0	0	0	0	0
Glucose	0	0	12	0	0	0	0	0	0
Hyaluronic Acid	0	0	0	12	0	0	0	0	0
MilliQ	0	0	0	0	12	0	0	0	0
NaCl	0	0	0	0	0	12	0	0	0
Nimenrix	0	0	0	0	0	0	12	0	0
Tap water	0	0	0	0	0	0	0	12	0
Water for injection	0	0	0	0	8	0	0	1	3

Supplementary Table 22. Nimenrix confusion matrix using the full-data (VITEK MS).

Falsified constituents and genuine vaccine	Amikacin	Gentamicin	Glucose	Hyaluronic acid	MilliQ	NaCl	Nimenrix	Tap water	Water for injection
Amikacin	10	0	0	0	0	0	0	0	0
Gentamicin	0	10	0	0	0	0	0	0	0
Glucose	0	0	10	0	0	0	0	0	0
Hyaluronic Acid	0	0	0	10	0	0	0	0	0
MilliQ	0	0	0	0	0	0	0	0	10
NaCl	0	0	0	0	0	10	0	0	0
Nimenrix	0	0	0	0	0	0	10	0	0
Tap water	0	0	0	0	0	0	0	10	0
Water for injection	0	0	0	0	0	0	0	0	10

Supplementary Table 23. Nimenrix confusion matrix using the training-data (VITEK MS).

Falsified constituents and genuine vaccine	Amikacin	Gentamicin	Glucose	Hyaluronic acid	MilliQ	NaCl	Nimenrix	Tap water	Water for injection
Amikacin	2	0	0	0	0	0	0	0	0
Gentamicin	0	2	0	0	0	0	0	0	0
Glucose	0	0	2	0	0	0	0	0	0
Hyaluronic Acid	0	0	0	2	0	0	0	0	0
MilliQ	0	0	0	0	0	0	0	0	2
NaCl	0	0	0	0	0	2	0	0	0
Nimenrix	0	0	0	0	0	0	2	0	0
Tap water	0	0	0	0	0	0	0	2	0
Water for injection	0	0	0	0	0	0	0	1	1

Supplementary Table 24. Nimenrix confusion matrix using the test-set data (VITEK MS).

Falsified constituents and genuine vaccine	Amikacin	Gentamicin	Glucose	Hyaluronic acid	Ixiaro	MilliQ	NaCl	Tap water	Water for injection
Amikacin	11	0	0	0	0	0	1	0	0
Gentamicin	0	12	0	0	0	0	0	0	0
Glucose	0	0	12	0	0	0	0	0	0
Hyaluronic Acid	0	0	0	12	0	0	0	0	0
Ixiaro	0	0	0	0	12	0	0	0	0
MilliQ	0	0	0	0	0	12	0	0	0
NaCl	0	0	0	0	0	0	12	0	0
Tap water	0	0	0	0	0	0	0	12	0
Water for injection	0	0	0	0	0	8	0	0	4

Supplementary Table 25. Ixiaro confusion matrix using the all-data (VITEK MS).

Falsified constituents and genuine vaccine	Amikacin	Gentamicin	Glucose	Hyaluronic acid	Ixiaro	MilliQ	NaCl	Tap water	Water for injection
Amikacin	7	0	1	0	0	0	2	0	0
Gentamicin	0	10	0	0	0	0	0	0	0
Glucose	0	0	10	0	0	0	0	0	0
Hyaluronic Acid	0	0	0	10	0	0	0	0	0
Ixiaro	0	0	0	0	10	0	0	0	0
MilliQ	0	0	0	0	0	0	0	0	10
NaCl	0	0	0	0	0	0	10	0	0
Tap water	0	0	0	0	0	0	0	10	0
Water for injection	0	0	0	0	0	0	0	0	10

Supplementary Table 26. Ixiaro confusion matrix using the training-set data (VITEK MS).

Falsified constituents and genuine vaccine	Amikacin	Gentamicin	Glucose	Hyaluronic acid	Ixiaro	MilliQ	NaCl	Tap water	Water for injection
Amikacin	2	0	0	0	0	0	0	0	0
Gentamicin	0	2	0	0	0	0	0	0	0
Glucose	0	0	2	0	0	0	0	0	0
Hyaluronic Acid	0	0	0	2	0	0	0	0	0
Ixiaro	0	0	0	0	2	0	0	0	0
MilliQ	0	0	0	0	0	0	0	0	2
NaCl	0	0	0	0	0	0	2	0	0
Tap water	0	0	0	0	0	0	0	2	0
Water for injection	0	0	0	0	0	0	0	1	1

Supplementary Table 27. Ixiaro confusion matrix using the test-set data (VITEK MS).

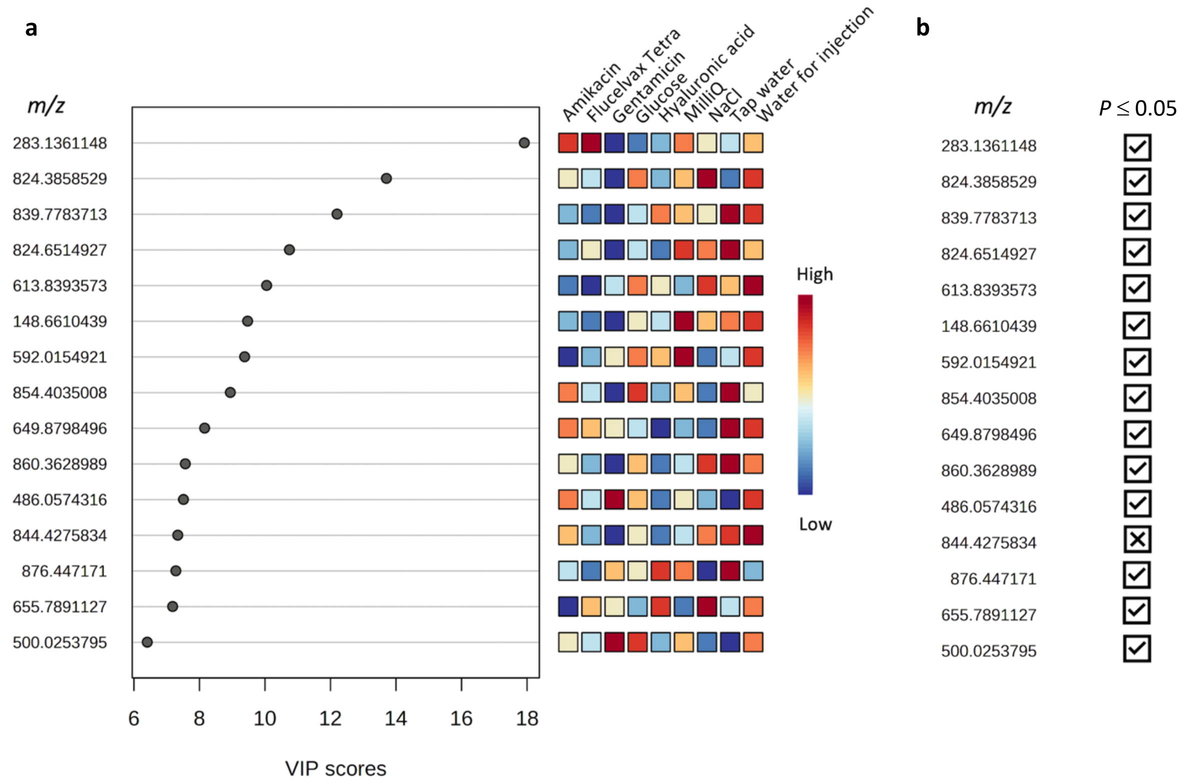

Supplementary Figure 16. Biotyper Sirius analyses of compound feature significance. (a) Variable importance in the projection (VIP) of the peaks at 0–900 *m/z* for Flucelvax Tetra vaccine compared to the 8 falsified constituents. The top 15 *m/z* values are plotted based on their VIP score. The heatmaps to the right of the plot represents the relative intensities of the *m/z* values for each sample group averaged over the group. (b) The two-way analysis of variance with Dunnett multiple comparison test results for the top 15 *m/z* values from the VIP analysis. *m/z* values with at least one statistically significant comparison $(P \leq 0.05)$ for a falsified constituent compared to Flucelvax Tetra are marked with a check. *m/z* values without a statistically significant comparison $(P > 0.05)$ are marked with an 'X' and are likely contributors to the VIP plot as important features for distinguishing between falsified constituents as opposed to distinguishing the genuine vaccine from the falsified constituents.

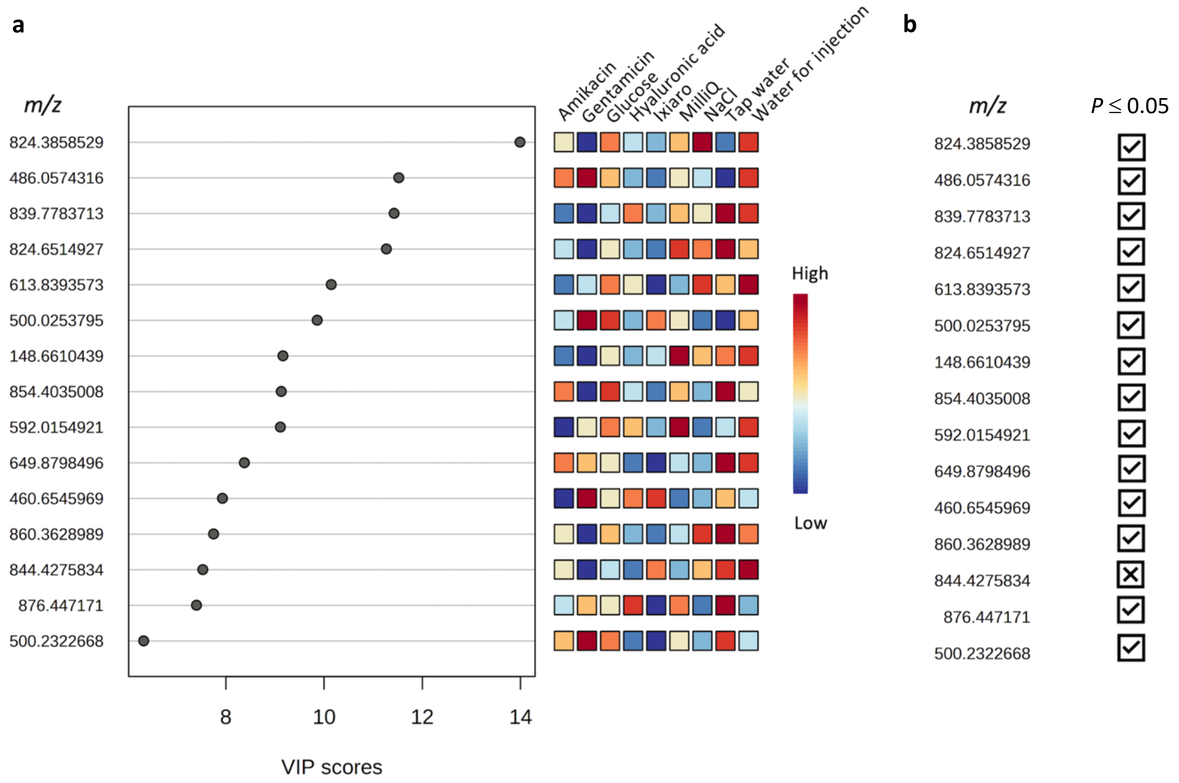

Supplementary Figure 17. Biotyper Sirius analyses of compound feature significance. (a) Variable importance in the projection (VIP) of the peaks at 0–900 *m/z* for Ixiaro vaccine compared to the 8 falsified constituents. The top 15 *m/z* values are plotted based on their VIP score. The heatmaps to the right of the plot represents the relative intensities of the *m/z* values for each sample group averaged over the group. (b) The two-way analysis of variance with Dunnett multiple comparison test results for the top 15 *m/z* values from the VIP analysis. *m/z* values with at least one statistically significant comparison ($P \leq 0.05$) for a falsified constituent compared to Ixiaro are marked with a check. *m/z* values without a statistically significant comparison ($P > 0.05$) are marked with an 'X' and are likely contributors to the VIP plot as important features for distinguishing between falsified constituents as opposed to distinguishing the genuine vaccine from the falsified constituents.

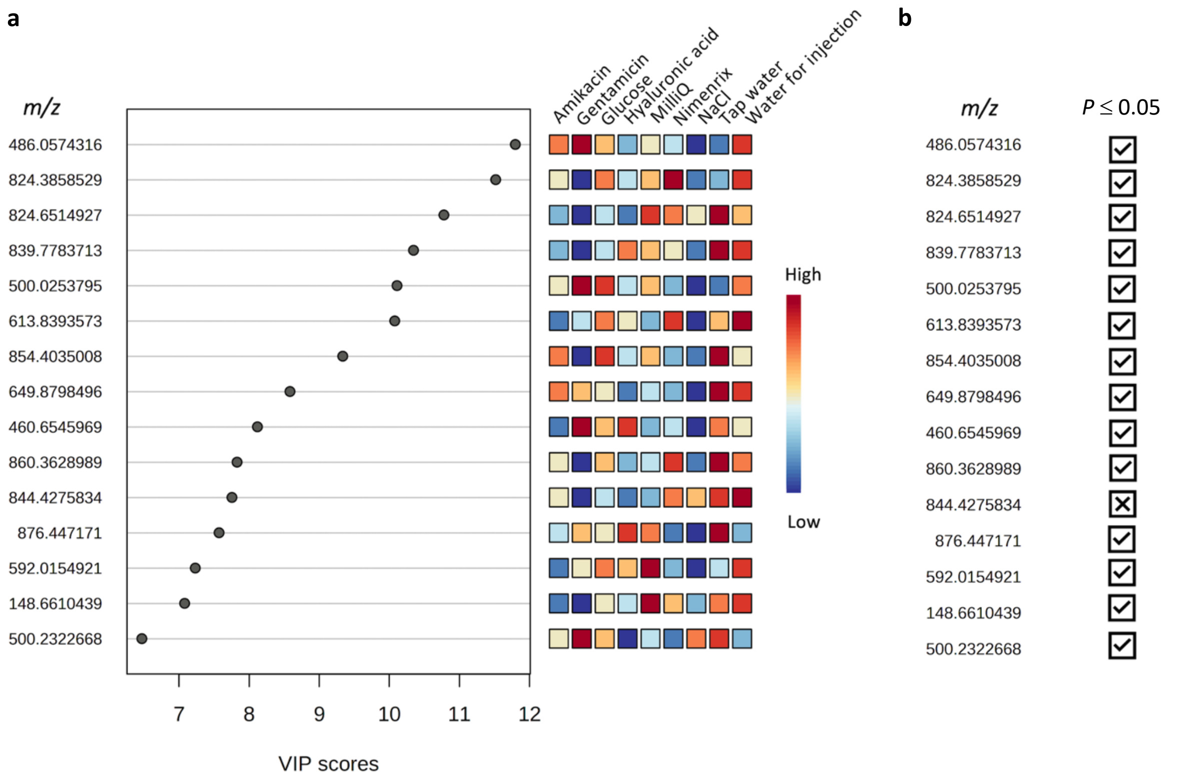

Supplementary Figure 18. Biotyper Sirius analyses of compound feature significance. (a) Variable importance in the projection (VIP) of the peaks at 0–900 *m/z* for Nimenrix vaccine compared to the 8 falsified constituents. The top 15 *m/z* values are plotted based on their VIP score. The heatmaps to the right of the plot represents the relative intensities of the *m/z* values for each sample group averaged over the group. (b) The two-way analysis of variance with Dunnett multiple comparison test results for the top 15 *m/z* values from the VIP analysis. *m/z* values with at least one statistically significant comparison ($P \leq 0.05$) for a falsified constituent compared to Nimenrix are marked with a check. *m/z* values without a statistically significant comparison ($P > 0.05$) are marked with an 'X' and are likely contributors to the VIP plot as important features for distinguishing between falsified constituents as opposed to distinguishing the genuine vaccine from the falsified constituents.

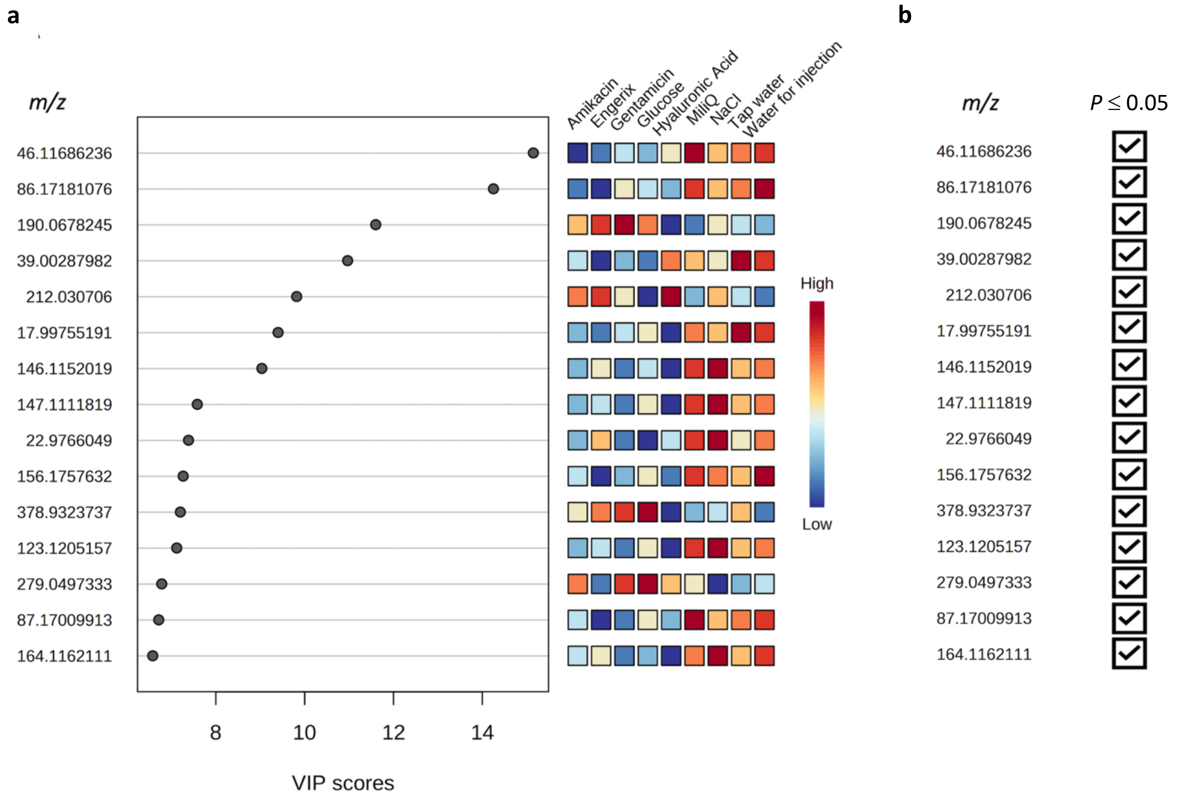

Supplementary Figure 19. VITEK MS analyses of compound feature significance. (a) Variable importance in the projection (VIP) of the peaks at 0–900 *m/z* for Engerix B vaccine compared to the 8 falsified constituents. The top 15 *m/z* values are plotted based on their VIP score. The heatmaps to the right of the plot represents the relative intensities of the *m/z* values for each sample group averaged over the group. (b) The two-way analysis of variance with Dunnett multiple comparison test results for the top 15 *m/z* values from the VIP analysis. *m/z* values with at least one statistically significant comparison *(P* ≤ 0.05) for a falsified constituent compared to Engerix B are marked with a check.

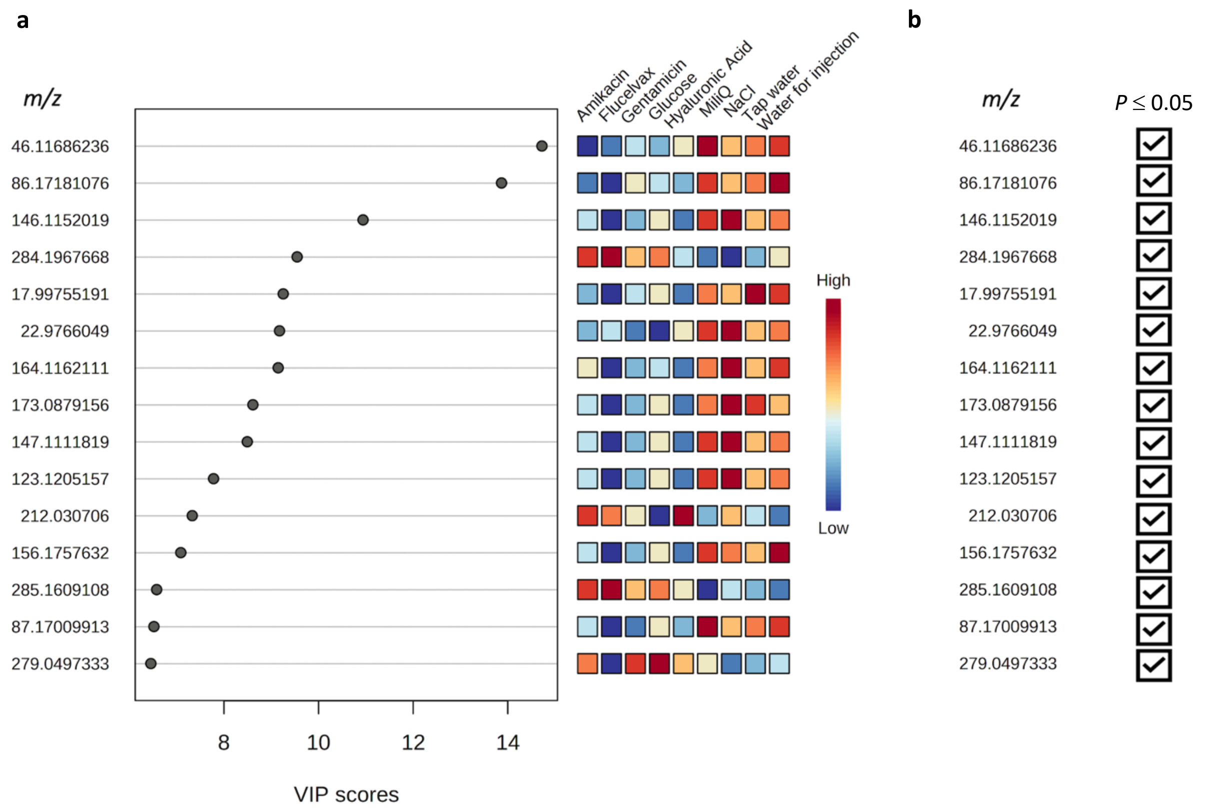

Supplementary Figure 20. VITEK MS analyses of compound feature significance. (a) Variable importance in the projection (VIP) of the peaks at 0–900 *m/z* for Flucelvax Tetra vaccine compared to the 8 falsified constituents. The top 15 *m/z* values are plotted based on their VIP score. The heatmaps to the right of the plot represents the relative intensities of the *m/z* values for each sample group averaged over the group. (b) The two-way analysis of variance with Dunnett multiple comparison test results for the top 15 *m/z* values from the VIP analysis. *m/z* values with at least one statistically significant comparison ($P \leq 0.05$) for a falsified constituent compared to Flucelvax Tetra are marked with a check.

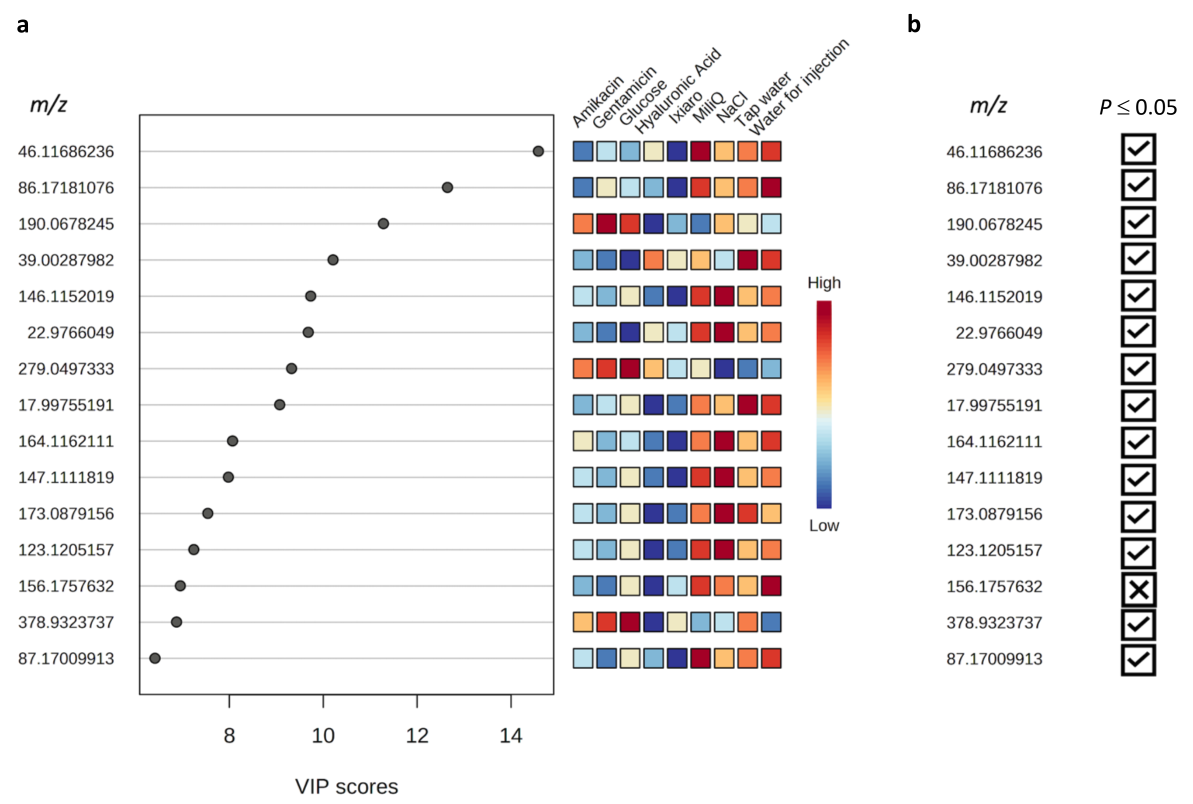

Supplementary Figure 21. VITEK MS analyses of compound feature significance. (a) Variable importance in the projection (VIP) of the peaks at 0–900 *m/z* for Ixiaro vaccine compared to the 8 falsified constituents. The top 15 *m/z* values are plotted based on their VIP score. The heatmaps to the right of the plot represents the relative intensities of the *m/z* values for each sample group averaged over the group. (b) The two-way analysis of variance with Dunnett multiple comparison test results for the top 15 *m/z* values from the VIP analysis. *m/z* values with at least one statistically significant comparison ($P \leq 0.05$) for a falsified constituent compared to Ixiaro are marked with a check. *m/z* values without a statistically significant comparison ($P > 0.05$) are marked with an 'X' and are likely contributors to the VIP plot as important features for distinguishing between falsified constituents as opposed to distinguishing the genuine vaccine from the falsified constituents.

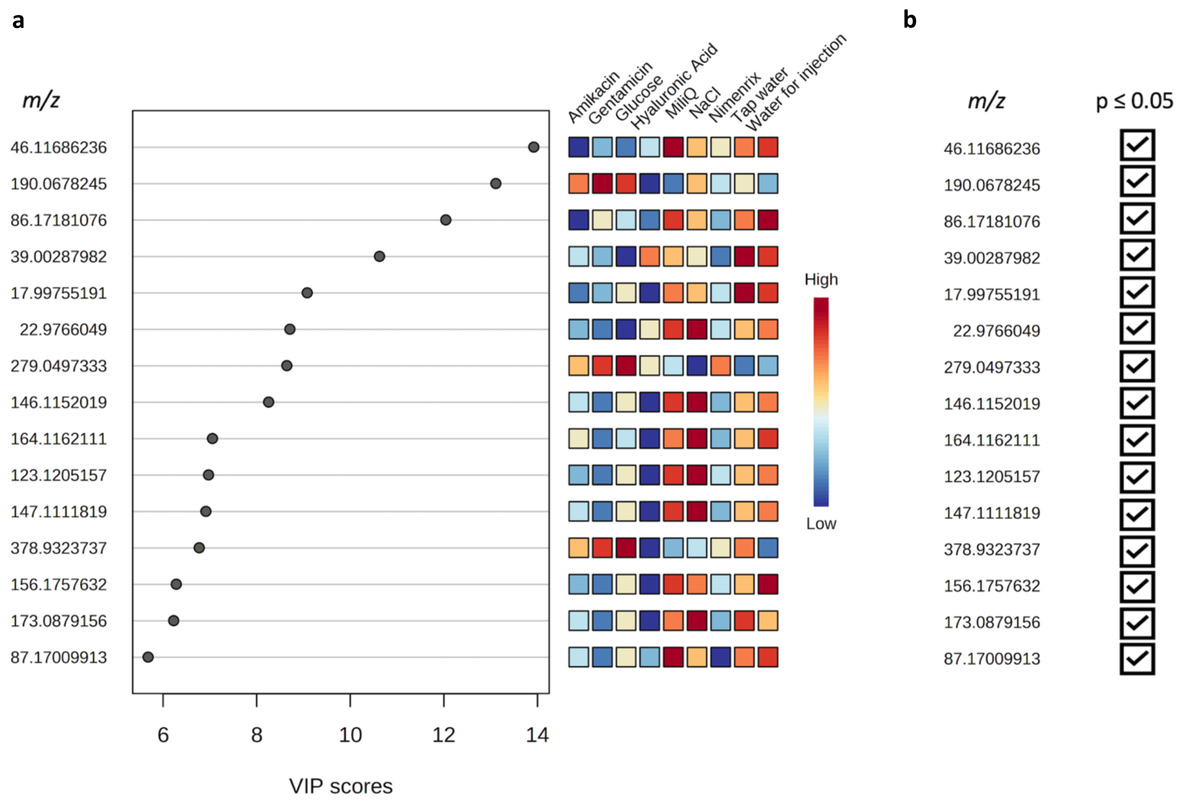

Supplementary Figure 22. VITEK MS analyses of compound feature significance. (a) Variable importance in the projection (VIP) of the peaks at 0–900 *m/z* for Nimenrix vaccine compared to the 8 falsified constituents. The top 15 *m/z* values are plotted based on their VIP score. The heatmaps to the right of the plot represents the relative intensities of the *m/z* values for each sample group averaged over the group. (b) The two-way analysis of variance with Dunnett multiple comparison test results for the top 15 *m/z* values from the VIP analysis. *m/z* values with at least one statistically significant comparison *(P* ≤ 0.05) for a falsified constituent compared to Nimenrix are marked with a check.

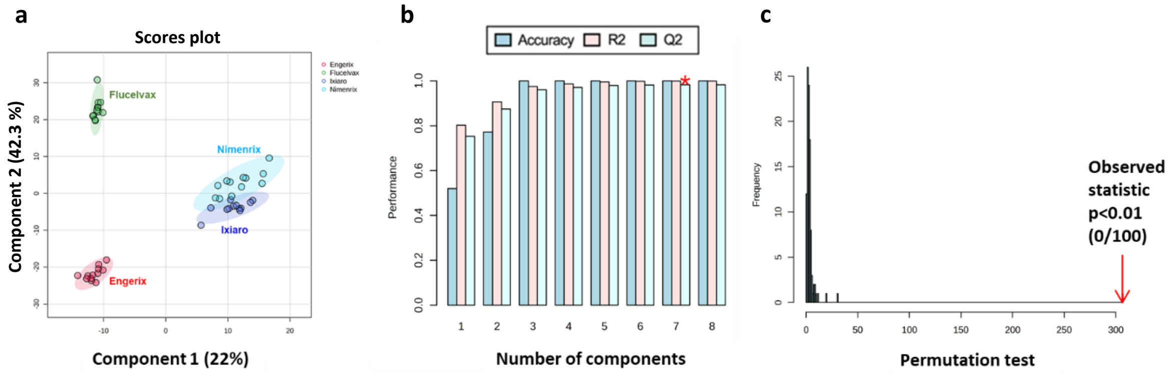

Supplementary Figure 23. VITEK MS partial least-squares discriminant analysis (PLS-DA) of authentic vaccines: Flucelvax Tetra, Nimenrix, Ixiaro and Engerix. (a) PLS-DA 2D scores plot shows samples group clustering. (b) Cross validation shows a minimum of 8 components (mass spectral peaks) are required to differentiate the experimental groups for the best Q2 value (shown by *). (c) Permutation testing showed the model was significant with p-value <0.01.
